# McTwo: a two-step feature selection algorithm based on maximal information coefficient

**DOI:** 10.1186/s12859-016-0990-0

**Published:** 2016-03-23

**Authors:** Ruiquan Ge, Manli Zhou, Youxi Luo, Qinghan Meng, Guoqin Mai, Dongli Ma, Guoqing Wang, Fengfeng Zhou

**Affiliations:** Shenzhen Institutes of Advanced Technology, and Key Lab for Health Informatics, Chinese Academy of Sciences, 1068 Xueyuan Avenue, Shenzhen University Town, Shenzhen, Guangdong 518055 P.R. China; Shenzhen College of Advanced Technology, University of Chinese Academy of Sciences, Shenzhen, Guangdong 518055 P.R. China; School of Science, Hubei University of Technology, Wuhan, Hubei 430068 P.R. China; Shenzhen Children’s Hospital, Shenzhen, Guangdong 518026 P.R. China; Department of Pathogenobiology, Basic Medical College of Jilin University, Changchun, Jilin China

**Keywords:** Maximal information coefficient (MIC), Heuristic algorithm, Feature selection, Filter algorithm, Wrapper algorithm

## Abstract

**Background:**

High-throughput bio-OMIC technologies are producing high-dimension data from bio-samples at an ever increasing rate, whereas the training sample number in a traditional experiment remains small due to various difficulties. This “large *p*, small *n*” paradigm in the area of biomedical “big data” may be at least partly solved by feature selection algorithms, which select only features significantly associated with phenotypes. Feature selection is an NP-hard problem. Due to the exponentially increased time requirement for finding the globally optimal solution, all the existing feature selection algorithms employ heuristic rules to find locally optimal solutions, and their solutions achieve different performances on different datasets.

**Results:**

This work describes a feature selection algorithm based on a recently published correlation measurement, Maximal Information Coefficient (MIC). The proposed algorithm, McTwo, aims to select features associated with phenotypes, independently of each other, and achieving high classification performance of the nearest neighbor algorithm. Based on the comparative study of 17 datasets, McTwo performs about as well as or better than existing algorithms, with significantly reduced numbers of selected features. The features selected by McTwo also appear to have particular biomedical relevance to the phenotypes from the literature.

**Conclusion:**

McTwo selects a feature subset with very good classification performance, as well as a small feature number. So McTwo may represent a complementary feature selection algorithm for the high-dimensional biomedical datasets.

**Electronic supplementary material:**

The online version of this article (doi:10.1186/s12859-016-0990-0) contains supplementary material, which is available to authorized users.

## Background

Due to the difficulty of collecting specific sample types and the limited available resources, only a small number of samples can be collected for a traditional biological study. However with modern biotechnologies huge amounts of biomedical“big data” may be produced for a single sample. This leads to the challenge of the“large *p* small *n*” paradigm in biological big data [1] which cannot be solved by the widely used deep learning strategy employed in other big data areas [2]. A “large *p* small *n*” dataset usually has dozens or at most a few hundred samples and millions or more features for each sample [1–3]. Over-fitting will be induced if all the features are used in the modeling of classification or regression for these samples [3]. One of the solutions is feature selection or dimension reduction, which tries to find a subset of features with the best modeling performance [3].

Various feature selection algorithms have been published, and they may be roughly grouped into three classes, based on how they determine the chosen features [4–6]. A class I wrapper algorithm usually adopts an existing data mining algorithm to evaluate a feature subset, and applies a heuristic feature screening rule for the feature subset with the optimal data mining performance. It tends to consume exponentially increased time to find such a feature subset. Class I algorithms usually use heuristic rules to find locally optimal solutions. The Prediction Analysis for Microarrays (PAM) [7] algorithm calculates a centroid for each of the class labels, and selects features to shrink the gene centroids toward the overall class centroid. PAM is robust for outlier features. The Regularized Random Forest (RRF) [8] algorithm uses a greedy rule by evaluating features on a subset of the training data at each random forest node. The choice of a new feature will be penalized if its information gain does not improve that of the chosen features.

A class II filter algorithm measures the association of each feature or feature subset with the sample labels, and orders all the features or feature subsets based on this measurement. Most of the filter algorithms evaluate the individual features. For the feature-based filter algorithms, the user has the option of deciding the number of top-ranked features for further experimental validations, but no information is provided for the feature subset with the optimal modeling performance. A filter algorithm does not consider the inter-feature correlations, but its linear calculation time complexity sometimes makes it the only affordable choice for large datasets [6]. *T*-test based filtering (TRank) algorithm is the most commonly used method to test for the difference of a feature between two groups. It estimates the difference between the two groups and the variation in the dataset giving a statistical significance measurement [9]. Wilcoxon test based feature filtering (WRank) algorithm calculates a non-parametric score of how discriminative a feature is between two classes of samples, and is known for its robustness for outliers [10]. ROC plot based filtering (ROCRank) algorithm evaluates how significant the Area Under the ROC Curve (AUC) of a feature is for the investigated binary classification performance [11]. The Correlation-based Feature Selection (CFS) [12] algorithm is a filter-based subset evaluation heuristic algorithm which assumes that features in a good feature subset should be independent of each other and are highly correlated with the samples’ class labels.

A class III hybrid algorithm aims to automatically generate an optimally selected feature subset by integrating the wrapper and filter strategies within different heuristic feature selection steps [6]. For example, Xing, et al. proposed a hybrid of filter and wrapper approaches to select a feature subset of a high-dimensional microarray dataset, and outperforms the regularization strategy with satisfactory classification error rates [13].

This study proposes a novel wrapper feature selection algorithm, McTwo, based on the measurement Maximal Information Coefficient (MIC) [14] between two variables. The first step of McTwo screens all the features for their MIC associations with the class labels and each other, and only those with significant discriminative power are kept for further screening. Then McTwo employs the best first search strategy to find the feature subset with the optimal classification performance. The experimental data suggests that this algorithm outperforms the other algorithms in most cases, with significantly reduced numbers of features.

## Methods

### The binary classification problem and its performance measurements

This work investigated the binary classification problem. A binary classification problem has two sets of samples, the Positive (*P*) and Negative (*N*) sets. *P* = {*P*_1_, *P*_2_, …, *P*_*n*_} and *N* = {*N*_1_, *N*_2_, …, *N*_*m*_}. The numbers of positive and negatives samples are also abbreviated as *P* = *n* and *N* = *m*, respectively. The total number of samples is *s* = *n* + *m*. Each sample *X*∈*P*∪*N* is a *k*-feature vector *X* = <*F*_1_(*X*), *F*_2_(*X*), … *F*_*k*_(*X*)>. A binary classifier f assigns *X* to either *P* or *N*.

Sensitivity (*Sn*), specificity (*Sp*) and accuracy (*Acc*) were widely used to measure how well a binary classification model performs [15–17]. Let *TP* and *FN* be the numbers of positive samples that are predicted by the model to be positive and negative, respectively. *TN* and *FP* are the numbers of negative samples, predicted to be negative and positive, respectively. So *P* = *TP* + *FN* and *N* = *TN* + *FP*. Sensitivity (*Sn*) is defined as the ratio of positive samples that are correctly predicted *Sn* = *TP*/(*TP* + *FN*) = *TP*/*P*, and specificity (*Sp*) is the ratio of corrected predicted negative samples *Sp* = *TN*/(*TN* + *FP*) = *TN*/*N*. The model’s overall accuracy is defined as *Acc* = (*TP* + *TN*)/(*TP* + *FN* + *TN* + *FP*) = (*TP* + *TN*)/(*P* + *N*) [18]. Another measurement *Avc* is defined as (*Sn* + *Sp*)/2 to help evaluate the unbalanced datasets with different numbers of positive and negative samples.

All the classification algorithms were evaluated for their overall performance measurements using 5 fold internal cross validations, averaged over 30 runs with different seeds for the random number generators. A binary classification algorithm with the larger *Acc* value performs better. If two models perform similarly well, the simpler model is preferred, since it costs less resource and human labour in its clinical deployment [15]. Also, a simpler model may avoid the over-fitting challenge in the biomedical big data area, caused by the “large *p* small *n*” paradigm [19]. External cross validations are also conducted to test whether McTwo generates feature selection bias.

The proposed feature selection algorithm may select features for any binary classification datasets. For the convenience of discussion and dataset availability, this study focuses on the classification performance comparison on the microarray-based gene expression profiling datasets.

### Biomedical datasets used in this study

Seventeen binary classification datasets were used for the classification performance evaluation in this study, as shown in Table 1. Two widely investigated datasets *Colon* [20] and *Leukaemia* [21] were retrieved from the R/Bioconductor packages *colonCA* and *golubEsets*, respectively. Six publicly available datasets, i.e. DLBCL [22], Prostate [23], ALL [24], CNS [25], Lymphoma [26] and Adenoma [27], were downloaded from the Broad Institute Genome Data Analysis Center, which is available at http://www.broadinstitute.org/cgi-bin/cancer/datasets.cgi. The dataset ALL was further processed into four binary classification datasets, i.e. ALL1, ALL2, ALL3 and ALL4, based on different phenotype annotations as described in Table 1. Another five new datasets, i.e. Myeloma (accession: GDS531) [28], Gastric (accession: GSE37023) [29], Gastric1/Gastric2 (accession: GSE29272) [30], T1D (accession: GSE35725) [31] and Stroke (accession: GSE22255) [32], were downloaded from the NCBI Gene Expression Omnibus (GEO) database.

**Table 1:** Summary of the 17 binary classification datasets used in this study

ID	Dataset	Samples	Features	Summary
1	DLBCL	77	7129	DLBCL patients (58) and follicular lymphoma (19)
2	Pros (Prostate)	102	12625	prostate (52) and non-prostate (50)
3	Colon	62	2000	tumour (40) and normal (22)
4	Leuk (Leukaemia)	72	7129	ALL (47) and AML (25)
5	Mye (Myeloma)	173	12625	presence (137) and absence (36) of focallesions of bone
6	ALL1	128	12625	B-cell (95) and T-cell (33)
7	ALL2	100	12625	Patients that did (65) and did not (35) relapse
8	ALL3	125	12625	with (24) and without (101) multidrug resistance
9	ALL4	93	12625	with (26) and without (67) the t(9;22) chromosome translocation
10	CNS	60	7129	medulloblastoma survivors (39) and treatment failures (21)
11	Lym (Lymphoma)	45	4026	germinalcentre (22) and activated B-like DLBCL (23)
12	Adeno (Adenoma)	36	7457	colon adenocarcinoma (18) and normal (18)
13	Gas (Gastric)	65	22645	tumors (29) and non-malignants (36)
14	Gas1 (Gastric1)	144	22283	non-cardia (72) of gastric and normal (72)
15	Gas2 (Gastric2)	124	22283	cardia (62) of gastric and normal (62)
16	T1D	101	54675	T1D (57) and healthy control (44)
17	Stroke	40	54675	ischemic stroke (20) and control (20)

Column “Dataset” gives the dataset names that will be used throughout this manuscript. Columns “Samples” and “Features” are the numbers of samples and features in this dataset, respectively. Column “Summary” describes the two sample classes, and the sample number in each class is given in the parenthesis. Details of the dataset and the original study may be found in the references listed in the column “Reference”

The raw data from the NCBI GEO database were normalized into the gene expression matrix with the default parameters of the RMA algorithm [33], and all the other datasets were downloaded as the normalized data matrix.

All the datasets used in this study are previously published by the other researchers, and publicly available, as described above. So neither ethics nor informed consent forms are needed from this study.

### Maximal information coefficient based feature screening (McOne)

Maximal information coefficient (MIC) tests the dependence between two variables and whether they have a linear or other functional relationship [14]. The measurement MIC is symmetric and normalized into a range [0, 1]. A high MIC value suggests a dependency between the investigated variables, whereas MIC = 0 describes the relationship between two independent variables. Although MIC seems equitable for different dependency types [34] and performs slightly worse than some other algorithms like the dynamic slicing algorithms and *t*-test [35], its ability to handle both numeric and category data will facilitate the future applications to heterogeneous biomedical datasets. The calculation function for MIC is implemented as the R package Minerva version 1.5 by the original authors. The statistical characterization of MIC and the comprehensive comparisons of MIC against the other statistical tests including Pearson correction and mutual information may be found in [14].

An MIC-based filtering step, *McOne*, is proposed to remove those features of little association with phenotypes or redundant with other features remaining in the feature subset, as described in the above pseudo-code. Firstly, a number of terms are defined. For a given binary classification problem, the class labels *C* = {*C*_1_, *C*_2_, …, *C*_*s*_}, *C*_*i*_∈{*P*, *N*}, and each sample has *k* features < *F*_1_(*X*), *F*_2_(*X*), … *F*_*k*_(*X*)>, where *F*_*j*_ is the *j*^*th*^ feature.

**Definition**: Information Relevant features: *S* = {*F*_*i*_ |MIC(*F*_*i*_, *C*) > *r*}, where *r* is a pre-set irrelevancy threshold.

**Definition**: Information Redundant features: *F*_*i*_ is redundant, if there exists another feature *F*_*j*_*, s.t.* MIC(*F*_*j*_,*C*) > MIC(*F*_*i*_,*C*) and MIC(*F*_*j*_,*F*_*i*_) > MIC(*F*_*i*_,*C*).

**Information dominant criterion**: Feature *F*_*j*_ will be kept, if it has the maximum information relevancy with target variable *C* in the candidate feature subset MIC(*F*_*j*_, *C*) and not redundant with the features already selected.

### McTwo algorithm

We employ the best first search strategy to further reduce the feature number. Our experimental data shows that McOne selects a subset of features with satisfying classification performances. However, McOne may select dozens, or even more than a hundred features, which may lead to the over-fitting problem for some big data areas with the “large *p* small *n*” challenge [3]. The best first search strategy is widely used for the purpose of further reducing the number of selected features in a small scale feature subset. This study uses the version implemented in the FSelector package version 0.19 in the software R version 3.0.2.

The *k* nearest neighbour (KNN) algorithm is used as the embedded classifier in the best first search procedure. Although KNN is a very simple classifier, its merits of fast computing and parameter independency make NN the ideal classifier when being executed many times in a wrap procedure. The simple form NN is chosen, i.e. *k* = 1.

The balanced accuracy *BAcc* = (*Sn* + *Sp*)/2 calculated by the leave-one-out (*LOO*) validation strategy is used as the optimization goal. This is because the overall accuracy *Acc* does not always reflect a reasonable classification performance of a classifier on an imbalanced dataset. For example, for a dataset of 100 positive and 10,000 negative samples, if a classifier predicts any samples as “negative”, it has *Acc* = 10,000/(100 + 10,000) = 99.01 %, but *Sn* = 0. In comparison, *BAcc* = (0 + 1)/2 = 0.5, which ranks such a classifier very low. Also, the LOO validation is parameter independent, and may be an objective strategy to evaluate how well a classifier performs.

The aforementioned step two of McTwo uses the output feature subset of McOne as its input, and returns the features filtered by the above procedure.

### Time intensity estimation of McTwo

Here is an estimation of the time complexity of the algorithm McTwo. McOne needs to calculate MIC values between all the features, and features against the class labels. Let *p* and *n* be the numbers of features and samples, respectively. So McOne runs for at most the time O(*p*^2^ + *p*), assuming that the MIC value between two variables is calculated within a constant time. The second step of McTwo theoretically needs to screen all the remaining features filtered by McOne, which is at most O(*p*). So the worst-case time complexity of McTwo is O(*p*^2^ + *p*) + O(*p*) ~ O(*p*^2^ + 2*p*) ~ O(*p*^2^), which is the same as the feature selection algorithm FCBF [36]. But the filtering step McOne is implemented to evaluate the MIC values between features and class labels, which will usually exclude most of the features. Then the evaluation of inter-feature MIC values will be significantly speeded up. So the actual calculation time will not reach the upper-bound O(*p*^2^) in most cases.

### Comparative analysis of feature selection performances

We conducted a series of comprehensive comparative experiments with the other commonly used feature selection algorithms, from both the classification accuracy and selected feature numbers aspects. The comparison was conducted against two wrapper algorithms (class I), i.e. PAM [7] and RRF [8], and three widely used filter algorithms (class II), i.e. TRank [9], WRank [10] and ROCRank [11]. Since the filter algorithm CFS automatically generates an optimally selected feature subset, CFS is grouped with the wrapper algorithms in the comparison experiments.

FCBF (Fast Correlation-Based Filter) [36] selects features in a similar way to McTwo. There are two major differences between FCBF and McTwo. In the first step, McTwo uses the measurement MIC to test the association between two variables [14], whereas FCBF uses the symmetric uncertainty (SU) based on information gain [36]. MIC is claimed to fit better on complex datasets than the other correlation measurements. In the second step, McTwo chooses the next feature based on the performance of an embedded classifier NN, while FCBF determines whether the next feature is kept by evaluating whether it, together with the existing features, may constitute an approximate Markov blanket, defined from the measurement SU. An external cross validation is conducted to compare the classification performances of the two algorithms.

A number of representative classification algorithms are chosen to build the binary classification models based on the features selected by the aforementioned feature selection algorithms. Support Vector Machine (SVM) calculates a hyper-plane between the two classes of samples/points in the high-dimensional space that maximizes the inter-class distance but minimizes the intra-class distances [37]. The Naive Bayes (NBayes) model assumes that the features are independent of each other and picks the class label with the maximal posterior probability as the prediction [38]. NBayes is known to be competitive with the more advanced and computationally-intensive methods, e.g. SVMs, in some machine learning problems such as text categorization [39]. A Decision Tree (DTree) consists of decision rules on the tree nodes about which route to take for the next decision step [40]. The simple Nearest Neighbour (NN) algorithm predicts that a query sample belongs to the same class as its nearest neighbour in a given distance measurement [41].

**Fig. 1:**
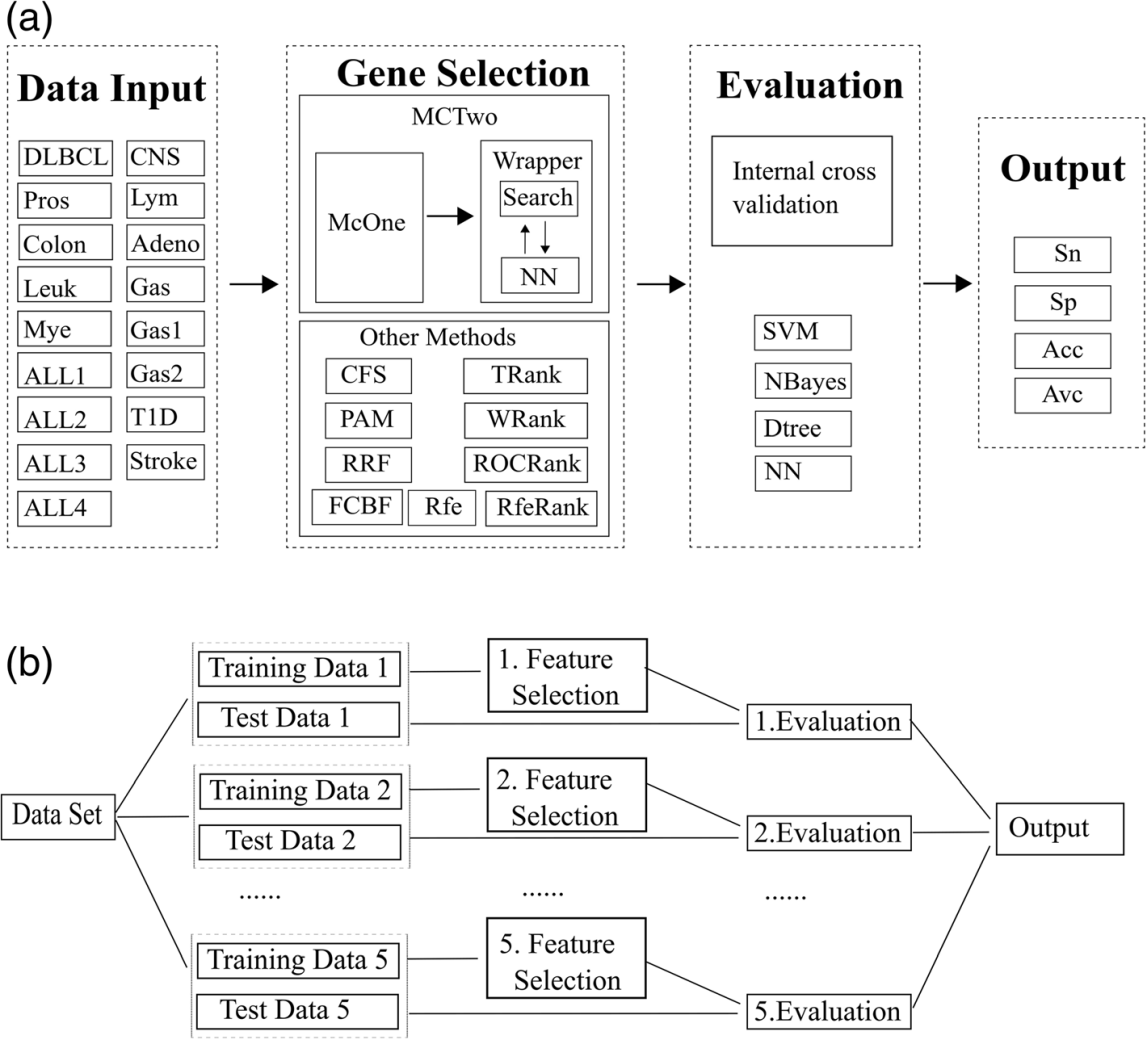
Experimental design of this study. There are 17 datasets used to evaluate the performances of the feature selection algorithms, as in box of “Data Input”. The structure of proposed McTwo algorithm is illustrated in the box “McTwo”. Nine other representative feature selection algorithms are listed in the box “Other Methods”. Four binary classification algorithms are used to evaluate what degree of accuracy the classification models based on the selected features may achieve. The classification performance is measured by the sensitivity (*Sn*), specificity (*Sp*), overall accuracy (*Acc*). **a** The processing scheme of internal cross validation. **b** The work flow of external cross validation

The whole procedure of the experiments is illustrated in Fig. 1.

## Results and discussion

### McTwo significantly decreases the feature number selected by McOne

The two datasets Gas1 and T1D are selected from the 17 datasets as representatives of cancers and cardiovascular diseases, respectively. The detailed results of all the other datasets can be found in Additional file 1: Figure S1. Results of all the 17 datasets will be summarized and discussed in the following text.

**Fig. 2:**
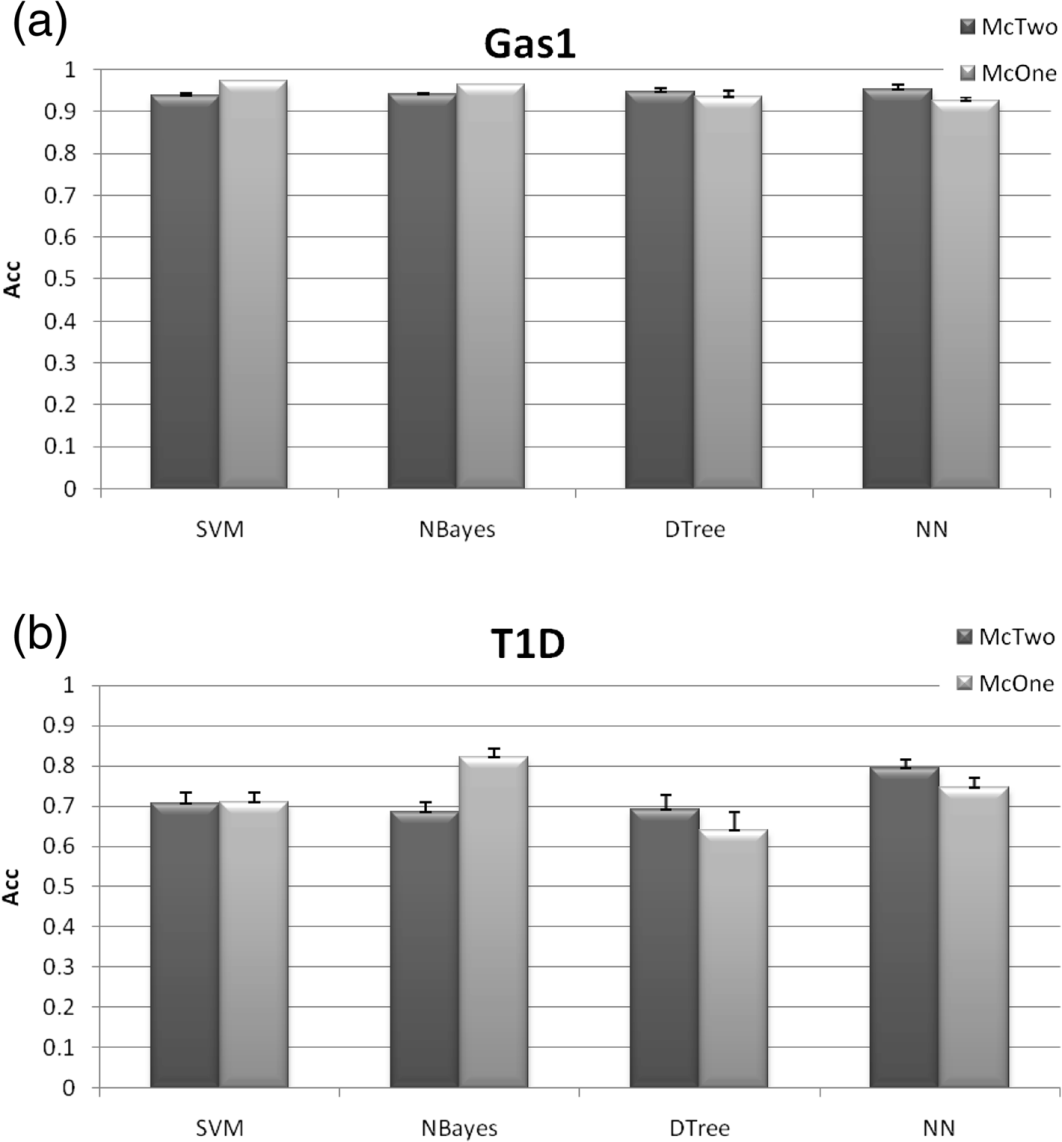
Comparison of the binary classification accuracy *Acc* between the two algorithms McTwo and McOne. The performance is illustrated on the two datasets **a** Gas1 and **b** T1D. Figures for the other datasets may be found in Additional file 1: Figure S1. The averaged value and the standard deviation of the classification *Acc* are calculated over the 30 runs of the 5-fold cross validations over the given dataset

McTwo achieves similar overall accuracies to McOne, using different classification algorithms, as shown in Fig. 2. Firstly, McOne outperforms McTwo only on one of the 17 datasets for the NN classification algorithm while on average McTwo outperforms McOne with a 3.99 % improvement in accuracy. This is within our expectation, since McTwo tries to minimize the feature number while keeping a similar overall classification accuracy in the second step. The only exception is the dataset Adeno, where McTwo has a 0.2 % smaller *Acc* (99.8 %) than McOne (100 %). But McTwo uses only 2 features to achieve almost similar classification performance as McOne’s 29-feature based model. On average, McTwo (90.99 %) outperforms McOne (86.99 %) in the overall accuracy *Acc* and the maximal improvement 10.6 % is achieved on the dataset Stroke. The DTree model of McTwo outperforms that of McOne for 14 out of the 17 datasets, the exceptions being Myeloma, ALL2 and CNS. The average improvement of McTwo over McOne is 3.4 %. But McOne outperforms McTwo with the averaged improvement in *A*cc of 3.00 and 4.86 % for the SVM and NBayes algorithms, respectively. This may be due to the fact that SVM [42] and NBayes [43] tend to be sensitive to the feature numbers, while McTwo selects a significantly smaller number of features than McOne, which will be discussed in the following paragraphs.

**Fig. 3:**
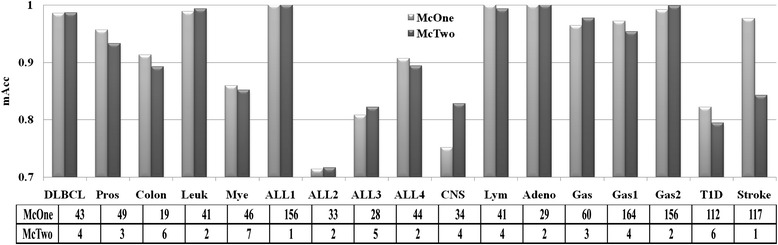
Comparison of the maximal classification accuracies and the feature numbers between McTwo and McOne. The two curves give the maximal classification accuracies, and the embedded table gives the feature numbers selected by McTwo and McOne for each of the 17 datasets

McTwo performs slightly worse in the best classification models than McOne, as shown in Fig. 3. For a given feature subset, researchers will always choose the classification model with the maximal overall accuracy. So the maximal *Acc* (*mAcc*) of the four classification algorithms (SVM, NBayes, DTree and NN) is used as the performance measurement of the feature subset selected by McTwo and McOne. Figure 3 shows that McTwo has an 0.8 % loss on average in *mAcc* than McOne, but performs equally well or better for 11 out of the 17 datasets than McOne. The largest difference of *mAcc* is observed for the dataset Stroke, where McOne outperforms McTwo by 13.4 %. There is only 0.01 % difference in the averaged *mAcc* between the two feature selection algorithms for the other 16 datasets.

McTwo selects a significantly smaller number of features than McOne, as shown in Fig. 3. On average, McTwo selects only 1/33.3 number of features to achieve similar classification accuracy compared with McOne. The largest numbers of features selected by McTwo and McOne are 7 and 164, respectively. For the dataset ALL1, both McTwo and McOne achieve 100 % in *mAcc* with McTwo using only one feature, compared to the 156 features selected by McOne.

So both of the two steps, i.e. McOne and the wrapper, are important in McTwo for finding the optimal subset of features.

### Comparison with the wrapper FS algorithms

**Fig. 4:**
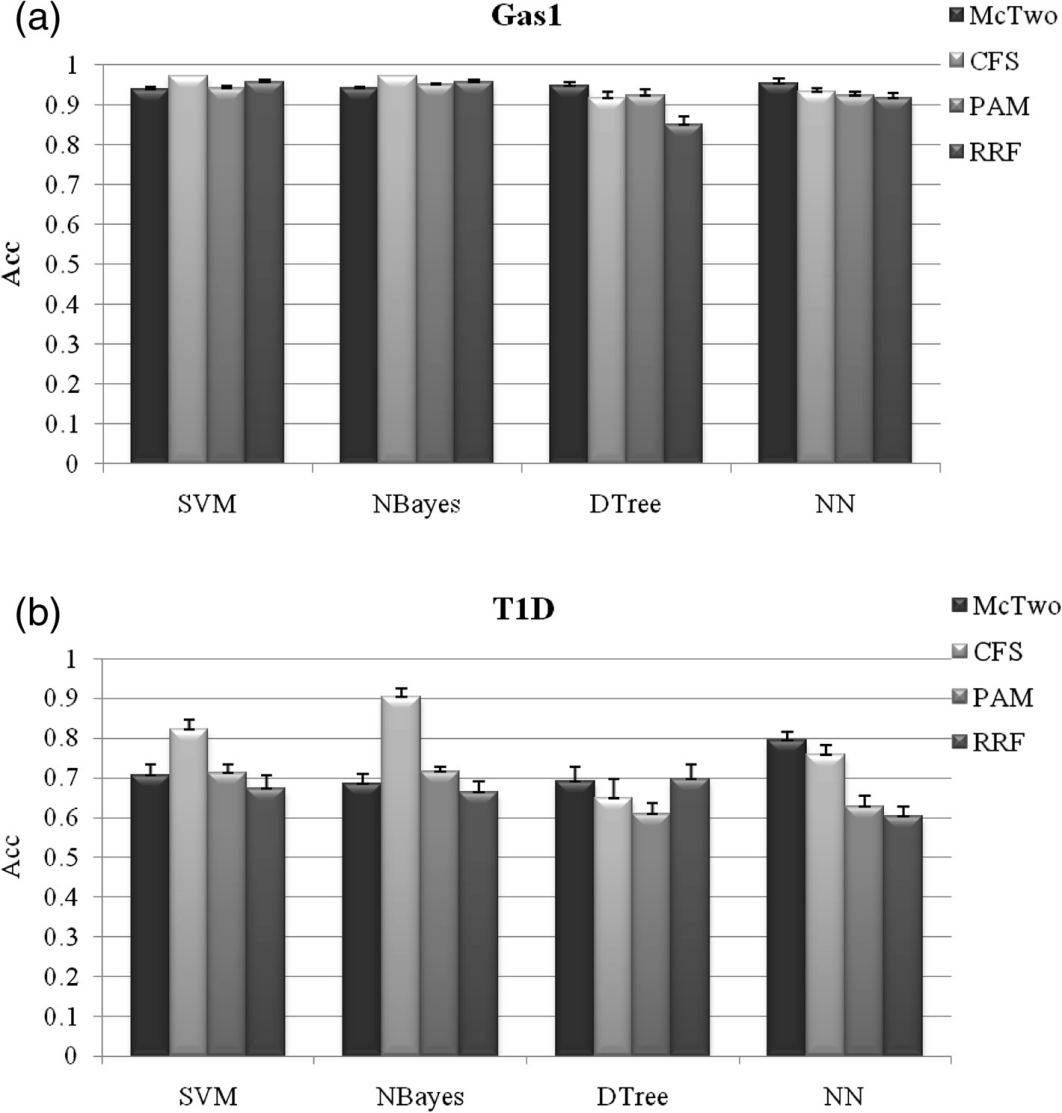
Comparison of the binary classification accuracy *Acc* among the four algorithms, McTwo, CFS, PAM and RRF. The performance is illustrated on the two datasets **a** Gastric1 and **b** T1D, and the figures for the other datasets may be found in Additional file 1: Figure S2. The averaged value and the standard deviation of the classification *Acc* are calculated over the 30 runs of the 5-fold cross validations over the given dataset

The classification performances of feature subsets selected by McTwo and three other wrapper algorithms CFS, PAM and RRF were compared. Best classification performance of the features selected by McTwo is usually achieved by the classification algorithms DTree and NN, as shown in Fig. 4 and Additional file 1: Figure S2.

McTwo performs much better than the algorithms PAM and RRF, but worse than CFS, as shown in Table 2. We use the comparison triplet win/tie/lose to measure the numbers of datasets that algorithm *A* performs better, equally well and worse compared with algorithm *B* by the measurement maximal accuracy *mAcc*, and this triplet is defined to be *CT*(*A*, *B*) = (win/tie/loss). McTwo performs better than PAM and RRF in 12 and 15 out of the 17 datasets, respectively. But McTwo does not achieve better *mAcc* than CFS in 14 datasets. It follows that CFS performs even better in *mAcc* compared with PAM and RRF, with CT(CFS, PAM) = (16/1/0) and CT(CFS, RRF) = (17/0/0).

**Table 2:** The comparison triplets between algorithm pairs from McTwo, CFS, PAM and RRF

CT(A, B)	McTwo	CFS	PAM	RRF
McTwo	0/17/0	**1/2/14**	12/1/4	15/0/2
CFS	**14/2/1**	**0/17/0**	**16/1/0**	**17/0/0**
PAM	4/1/12	**0/1/16**	0/17/0	13/0/4
RRF	2/0/15	**0/0/17**	4/0/13	0/17/0

The comparison triplet CT(A, B) is defined to be the numbers of the 17 datasets where algorithm A performs better, equally well and worse, compared with algorithm B. The measurement *mAcc* is used for comparison. The column and row of CFS are highlighted in bold

**Fig. 5:**
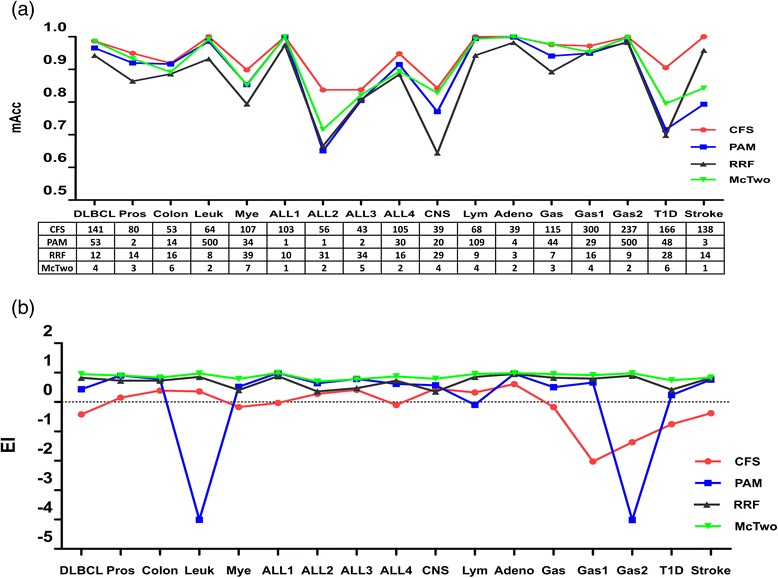
Should this be’Plots of classification accuracy and model complexity and of the combined measure EI. **a** The line plots of the classification model’s overall accuracy with the four wrapper algorithms on the 17 datasets, and the corresponding numbers of features selected by the different feature selection algorithms. **b** The line plots of the measurement EI. (Note that there is no relationship between the neighbouring datasets connected by the line which is simply included for convenience to identify data points for each algorithm. This is especially necessary where two algorithms have similar data)

The balance between the classification accuracy and the model complexity for the four wrapper algorithms was also investigated, as shown in Fig. 5. On average, as we have seen, McTwo achieves 3.5 % lower than CFS in *mAcc*, but 1.9 and 3.9 % better than PAM and RRF, respectively. But McTwo only needs 1/44.4 number of features on average compared with CFS. For example, both McTwo and CFS achieve 100 % in *mAcc* on dataset ALL1, but McTwo uses only one feature, compared with 103 features selected by CFS. There is currently no measure available to rate a classification model on both prediction accuracy and model complexity. Here we define an evaluation index of model complexity and classification accuracy *EI* = *Acc*-*p*/100 for this purpose, where *p* is the number of features used in the classification model. Except for the PAM feature selection algorithm on the dataset ALL3, McTwo performs best compared with all the other three wrapper algorithms on all the 17 datasets. McTwo performs worse than PAM in *Acc* on the four datasets Colon, Mye, ALL4 and Lym, with differences of 2.4, 0.2, 2.1 and 0.1 %, respectively. The comparison of feature numbers selected by McTwo and PAM for the four datasets shows that McTwo recommends significantly smaller numbers of features, i.e. (6 vs 14), (7 vs 34), (2 vs 30) and (4 vs 109), respectively. Similar observations may be found on the two datasets Gas1 and Stroke where McTwo performs worse than RRF. CFS and PAM also show a high fluctuation in the numbers of finally chosen features for different datasets, as shown in Fig. 5a.

Generally, McTwo outperforms PAM and RRF on both the classification accuracy and the model complexity. Although CFS slightly outperforms McTwo in the averaged measurement *mAcc*, McTwo uses significantly smaller numbers of features than CFS. Using the balanced model performance measurement EI, McTwo outperforms almost all the three wrapper algorithms on all the 17 datasets, as shown in Fig. 5b.

### Comparison with the filter FS algorithms

We further compare McTwo with the three filter algorithms TRank, WRank and ROCRank for their classification performances. A filter algorithm only outputs an ordered list of features based on a ranking measurement. So for a fair comparison, this study chooses top *p* features from the ordered list of features ranked by the filter algorithms, where *p* is the number of features chosen by McTwo.

**Fig. 6:**
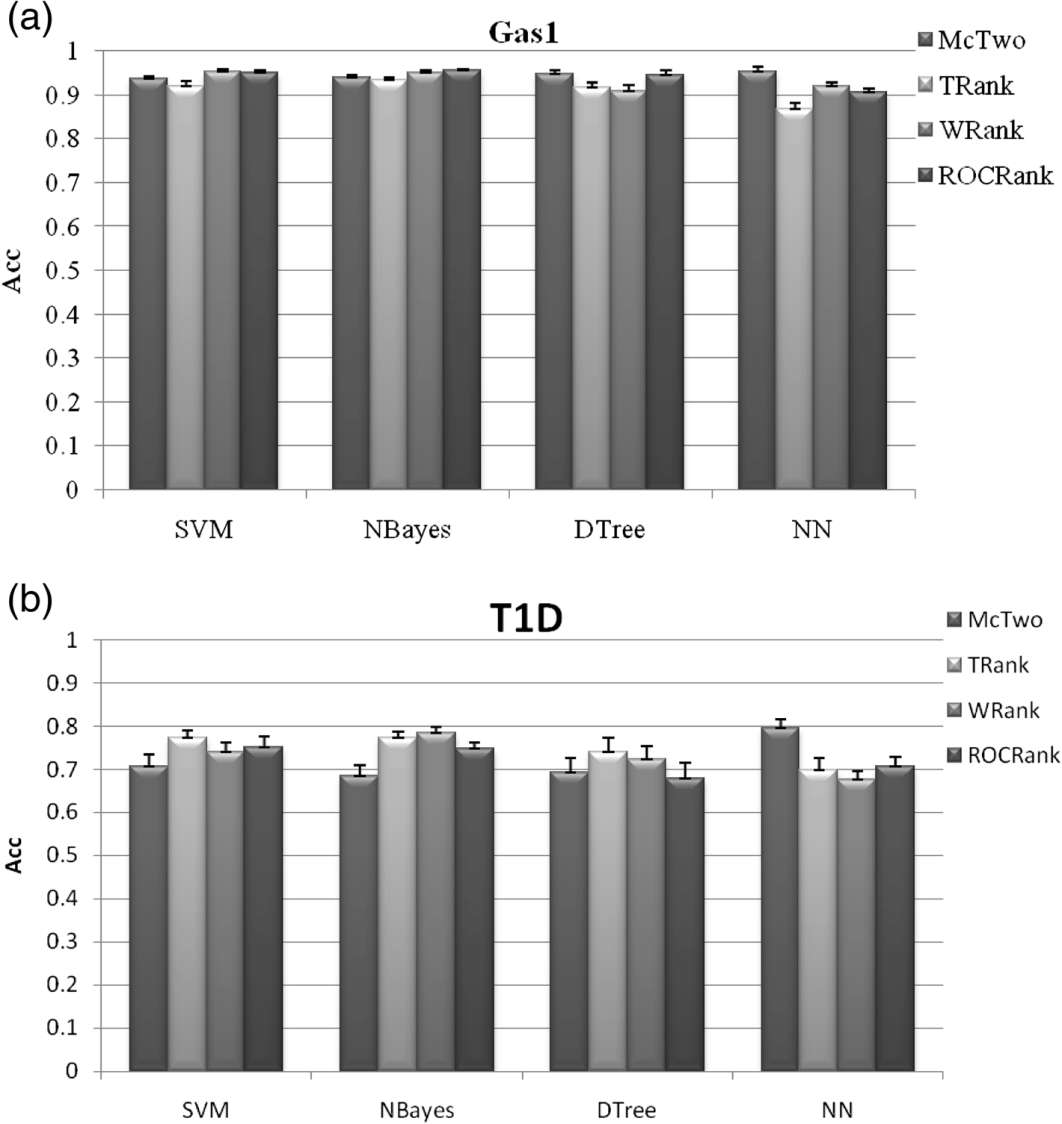
Comparison of the binary classification accuracy *Acc* among the four algorithms, McTwo, TRank, WRank and RCORank. The performance is illustrated on the two datasets **a** Gastric1 and **b** T1D. The figures for the other datasets may be found in Additional file 1: Figure S3. The averaged value and the standard deviation of the classification *Acc* are calculated over the 30 runs of the 5-fold cross validations over the given dataset

McTwo outperforms practically all the three other filter algorithms on 15 out of the 17 datasets, when using the NN classification algorithm. The only two exceptions are that ROCRank algorithm performs 0.8 and 0.2 % better than McTwo in *Acc* using NN on the dataset Pros and Adeno, respectively. The three other classification algorithms based on McTwo features perform similarly well or better compared with the classification performances based on the features of the three filter algorithms. Figure 6a shows that the best McTwo model using NN has an *Acc* 0.3 % smaller that of the best ROCRank model using NBayes on the dataset Gas1. For the dataset T1D, the NN classification model based on McTwo features outperforms almost all the other classification models. The one exception is that on the dataset ALL3 (0.7848), PAM outperforms McTwo (0.7720) with 0.0128 in *Acc*, as shown in Fig. 6b. The performance measurements *Sn*/*Sp*/*Acc*/*Avc* of all the 17 datasets are given in Additional file 1: Figure S3.

**Fig. 7:**
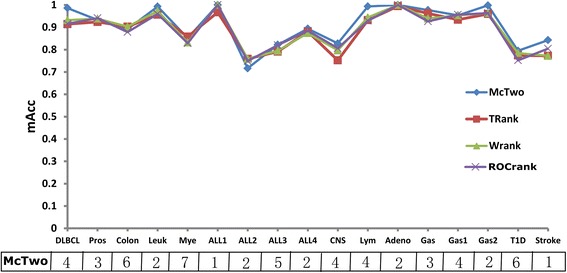
A combined plot of classification accuracy and model complexity. The line plots of the classification model’s overall accuracy of the four wrapper algorithms on the 17 datasets, and the corresponding numbers of features selected by the different feature selection algorithms. Note, there is no relationship between the neighbouring datasets connected by the line, and that is just for the convenience of find the dots for each algorithm, especially when two algorithms have similar data

McTwo and the three filter algorithms are compared pairwisely, and the results are illustrated using comparison triplets in Table 3. Firstly, McTwo performs as well as or better than the three filter algorithms on 14 datasets. The three filter algorithms outperform McTwo on three different datasets in the measurement *mAcc*. All the three filter algorithms, TRank (0.759), WRank (0.759) and ROCRank (0.749) outperform McTwo (0.716) on the dataset *ALL2*, as detailed in the Additional file 1: Table S2. ALL2 is the most difficult dataset for all four algorithms and the three wrapper algorithms (Figs. 5 and 7). CFS performs better on *mAcc* (0.837) but used 56 features compared to 0.716 for McTwo which selected only two features. In all the other cases the improved *mAcc* values of the filter algorithms is no more than 1.1 % better than with McTwo, as in Additional file 1: Table S2.

**Table 3:** The comparison triplets between algorithm pairs from McTwo, TRank, WRank and ROCRank

CT(A, B)	McTwo	TRank	WRank	ROCRank
McTwo	0/17/0	14/0/3	11/3/3	12/2/3
TRank	3/0/14	0/17/0	3/3/11	6/0/11
WRank	3/3/11	11/3/3	0/17/0	8/3/6
ROCRank	3/2/12	11/0/6	6/3/8	0/17/0

The comparison triplet CT(A, B) is defined to be the numbers of the 17 datasets where algorithm A performs better, equally well and worse, compared with algorithm B. The measurement *mAcc* is used for comparison

The above data demonstrates that McTwo performs better than the three filter algorithms on most of the 17 datasets, and similarly well on the others.

### External cross validations of the feature selection algorithms

Five-fold external cross validation is conducted for comparing McTwo with the other feature selection algorithms. Due to the excessive computation requirement of the CFS algorithm, the three largest datasets ALL1, Gas1 and Mye are chosen for the comparative study of external cross validations. External cross validations are recommended to evaluate whether a feature selection algorithm has a selection bias for small datasets [44–46]. The widely-used feature selection algorithm, i.e. Support Vector Machine based on Recursive Feature Elimination (SVM-RFE), may be used as either filter or wrapper model [47]. These are denoted as RfeRank and Rfe in this comparison, respectively.

**Fig. 8:**
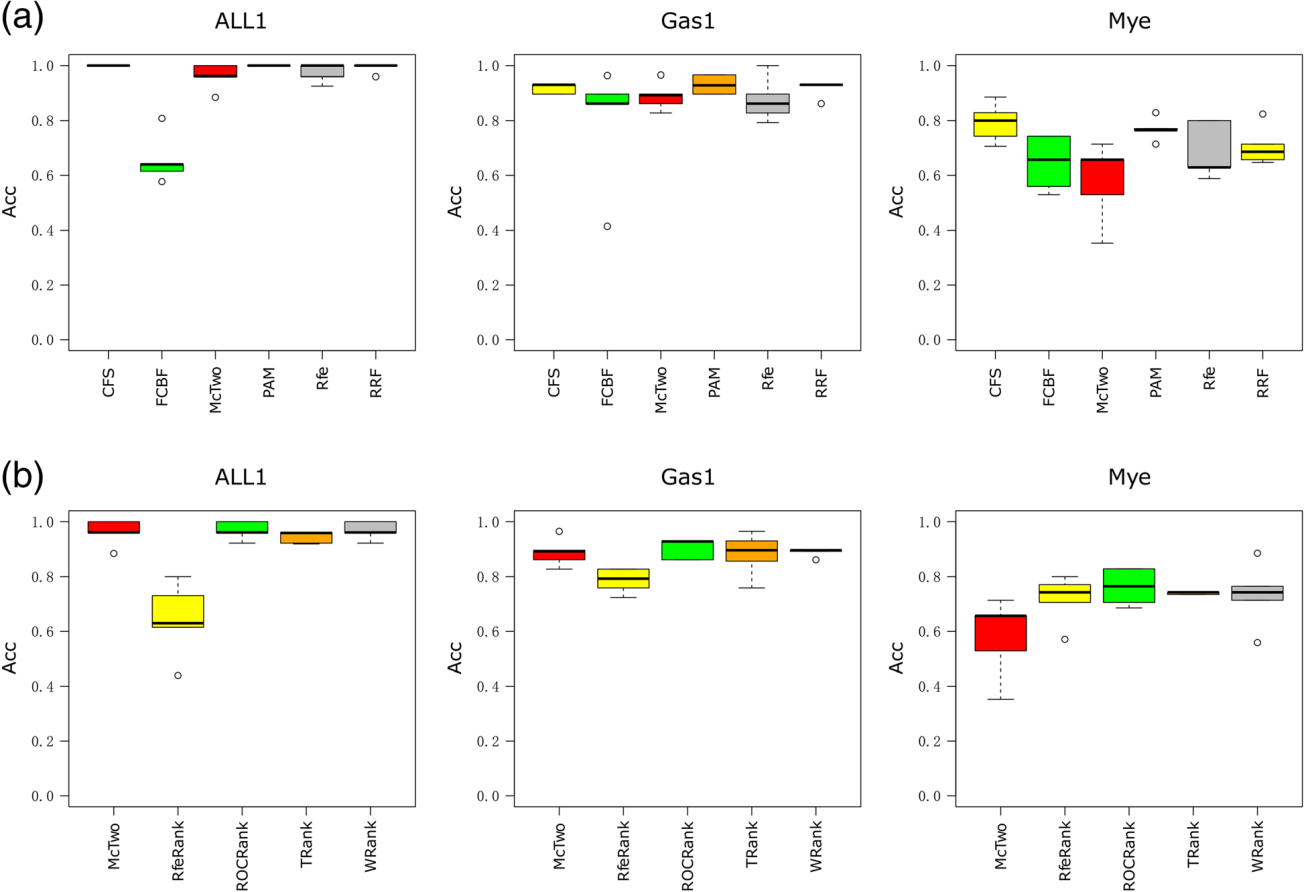
Boxplots of external cross validations of McTwo compared with the other feature selection algorithms. **a** Comparison among the six wrapper algorithms CFS, FCBF, McTwo, PAM, Rfe and RRF. **b** Comparison among the five filter algorithms McTwo, RfeRank, ROCRank, TRank and WRank

McTwo achieves satisfactory and stable classification performances using the external cross validations on the three investigated datasets, as shown in Fig. 8. As in the results of internal cross validations, McTwo does not achieve the best classification performances on the two datasets ALL1 (mAcc = 0.969) and Gas1 (mAcc = 0.903), but its performances are similar to those of the other algorithms. McTwo also shows much smaller variations compared with both wrapper and filter algorithms on the datasets ALL1 and Gas1. Similar low variations are only achieved by CFS, PAM, RRF and TRank on the dataset ALL1. The dataset Mye is challenging for all the feature selection algorithms, none of which achieve much in excess of 0.800. All the feature selection algorithms produce similar variations for the dataset Mye. McTwo has a similar feature screening outline to FCBF, but achieves better classification performances on the three investigated datasets. This is probably due to the fact that McTwo targets the classification performance as its optimization goal.

The statistical significance is also evaluated using the paired *t*-test to determine whether McTwo performs better than, similar to, or worse than each of the other feature selection algorithms [48]. The results are shown in Additional file 1: Table S3. For a confidence level 0.95, CFS and PAM perform statistically significantly better than McTwo on all the datasets. And McTwo performs similarly or slightly better than all the other wrapper algorithms. Except for the dataset Mye, McTwo performs better than all the investigated filter algorithms on all the datasets. When a slightly more stringent confidence level 0.99 is chosen, no feature selection algorithms perform better than McTwo except on the dataset Mye. The algorithms CFS and PAM perform better than McTwo with statistical significance. However McTwo uses only 1/3 as many features as CFS and PAM to achieve similar or just slightly worse classification performances.

### Best wrapper features are not always top-ranked by filter algorithms

As shown in Fig. 6, the best McTwo model performs similarly well to or better than the three filter feature selection algorithms, however the features selected are not always the top-ranked ones evaluated by the filter algorithms. Table 4 summarizes how each of the 4 features of Gastric1 and 6 features of T1D selected by McTwo is ranked by the three filter algorithms.

**Table 4:** The rankings of the features selected by McTwo from the three filter algorithms

Dataset	Probeset	TRank	WRank	ROCRank
Gastric1	216381_x_at	9	9	1
	209902_at	831	143	237
	205523_at	235	178	266
	218595_s_at	604	241	187
T1D	1560237_at	1450	1817	82
	1570327_at	17598	14048	41447
	208031_s_at	29	173	3921
	1569685_at	42946	40628	38453
	239925_at	23068	12694	35843
	1556521_a_at	32784	32691	52455

The probeset IDs are given in the column “Probeset”, and the rankings from the three ranking algorithms are in the last three columns, respectively

Except for that the probeset 216381_x_at is ranked as 9, 9 and 1 by TRank, WRank and ROCRank, respectively, all the other features selected by McTwo are ranked lower than 25, as shown in Table 4. For example, the dataset Gastric1 even has a 831-ranking feature selected into the classification model with 95.35 % in overall accuracy. The dataset T1D has 54,675 features, and the McTwo-based NN classification model outperforms all the other models in the overall accuracy, as shown in Fig. 6b. But this best model uses a feature ranked 52,455 out of the 54,675 features by ROCRank. A widely-used feature selection strategy based on the filter algorithms is to choose the top-*K* ranked features where *K* is usually determined by trial and error. So such low-ranked features will normally be removed by any filter algorithms.

Our data suggests that best classification models may use some features which are ranked low by filter algorithms. This is plausible as the filter algorithm evaluates the association of each feature with the class labels independently, and a combination of the top *p* ranked features does not necessarily lead to a classification model with high overall accuracy. For example, the features linearly correlated with the top ranked feature will also be highly ranked. However a combination of these linearly correlated highly-ranked features will not improve the classification model based on the top ranked feature. A lower-ranked feature independent of the top ranked feature may lead to a better classification model.

### Biological inferences of the McTwo selected features

Although most of the features selected by McTwo are ranked low by the filter algorithms, many have known roles in disease onset and development. For example two of the Gastric1 features, 216381_x_at and 218595_s_at, are known to be associated with gastric cancer, as shown in Table 4. Probeset 216381_x_at of the gene AKR7A3 (aldo-keto reductase family 7, member A3) is involved in the biological processes of cellular aldehyde metabolics and oxidation reduction. An independent study observed its differential transcriptional levels between gastric cancers and control samples [49]. Probeset 218595_s_at of the gene HEATR1 (HEAT repeat containing 1) may prevent apoptosis and induce gastric carcinoma in *Helicobacter pylori*-infected gastric epithelial cells [18].

Two other probesets 209902_at and 205523_at are extensively associated with many cancer types, but their association with gastric cancer needs to be further investigated [50–55]. Probeset 209902_at of the gene ATR (ataxia telangiectasia and Rad3 related; similar to ataxia telangiectasia and Rad3 related protein) is a serine/threonine protein kinase. ATR acts as a DNA damage sensor and activates checkpoint signals such as BRCA1, CHEK1, MCM2, RAD17, RPA2, and p53/TP53 when exposed to harmful influences such as IR (ionizing radiation) and UV (ultraviolet light). These conditions can lead to blocking DNA replication and mitosis, and promoting DNA repair and apoptosis. ATR is related to various types of cancers, such as esophageal adenocarcinoma, oropharyngeal cancer, endometrioid endometrial cancer, breast cancer, ovarian cancer and others [50–53]. Probeset 205523_at of the gene HAPLN1 (hyaluronan and proteoglycan link protein 1) can keep the polymerides of proteoglycan monomers and hyaluronic acid in the cartilage matrix. HAPLN1 is involved with biological process ranging from cell adhesion to biological adhesion. HAPLN1 is known to be associated with many cancer types, such as esophageal adenocarcinoma, breast cancer, colorectal cancer and others [54, 55]. A recent study shows that the over-expression of HAPLN1 and its SP-IgV domain improves tumorigenic properties of malignant pleural mesothelioma. Thus HAPLN1 may be of relevance for cancer treatment [56].

One of the six T1D features selected by McTwo, i.e. 208031_s_at, is also known to be closely associated with the development of diabetes. Probeset 208031_s_at of the gene RFX2 (regulatory factor X, 2 (influences HLA class II expression)) is a transcription factor. The transcriptional activator *rfx2* can bind to DNA in the promoter of the IL -5 receptor alpha gene. RFX2 is involved in the biological processes of transcription, regulation of transcription and regulation of RNA metabolism. It has been demonstrated that RFX2 plays an essential role in the development of diabetes in the DREAM (Diabetes Reduction Assessment with ramipril and rosiglitazone Medication) Study [57].

## Conclusions

This study describes a novel MIC-based wrapper feature selection algorithm, McTwo. McTwo achieves better or similar classification performances compared to the existing feature selection algorithms, and recommends a smaller number of features compared to the other wrapper algorithms. Using the same number of features, McTwo also achieves better or similar performance compared to other filter algorithms. The features selected by McTwo may lead to interesting biological hypotheses for further experimental investigation.

## Additional file

Additional file 1: Figure S1. Comparison of the binary classification accuracy Acc between the two algorithms McTwo and McOne. Figure S2. Comparison of the binary classification accuracy Acc among the four algorithms, McTwo, CFS, PAM and RRF. Figure S3. Comparison of the binary classification accuracy Acc among the four algorithms, McTwo, TRank, WRank and RCORank. Table S1. Comparison of the binary classification accuracy Acc between the two algorithms McTwo and McOne. Table S2. Comparison of McTwo with the three individual ranking algorithms. Table S3. Statistical significance of the comparison triplets of McTwo with the other feature selection algorithms. (PDF 931 kb)
