# Supplementary material for: McTwo: a two-step feature selection algorithm based on maximal information coefficient
Source: BMC Bioinformatics. 2016 Mar 23;17:142. doi: 10.1186/s12859-016-0990-0 (PMC4804474; doi:10.1186/s12859-016-0990-0)
Supplement: Additional file 1: Figure S1. — Comparison of the binary classification accuracy Acc between the two algorithms McTwo and McOne. Figure S2. Comparison of the binary classification accuracy Acc among the four algorithms, McTwo, CFS, PAM and RRF. Figure S3. Comparison of the binary classification accuracy Acc among the four algorithms, McTwo, TRank, WRank and RCORank. Table S1. Comparison of the binary classification accuracy Acc between the two algorithms McTwo and McOne. Table S2. Comparison of McTwo with the three individual ranking algorithms. Table S3. Statistical significance of the comparison triplets of McTwo with the other feature selection algorithms. (PDF 931 kb) [file 12859_2016_990_MOESM1_ESM.pdf]

McTwo: a two-step feature selection algorithm based on maximal information coefficient

Ruiquan Ge[1,2,*], Manli Zhou[1,2,*], Youxi Luo[1,3,*], Qinghan Meng[1,2], Guoqin Mai[1], Guoqing Wang[4,#], Fengfeng Zhou[1,#].

1. Shenzhen Institutes of Advanced Technology, and Key Lab for Health Informatics, Chinese Academy of Sciences, Shenzhen, Guangdong, 518055, P.R. China;
2. Shenzhen College of Advanced Technology, University of Chinese Academy of Sciences Shenzhen, Guangdong, P.R. China, 518055;
3. School of Science, Hubei University of Technology, Wuhan, Hubei, 430068, P.R. China.
4. Department of Pathogenobiology, Basic Medical College of Jilin University, Changchun, Jilin, China.

* These authors contribute equally to this work.
# Corresponding author: Fengfeng Zhou, phone: +86-755-86392200; fax: +86-755-86392299; e-mail: FengfengZhou@gmail.com, or ff.zhou@siat.ac.cn. Web site: http://www.healthinformaticslab.org/ffzhou/ . Postal address: 1068 Xueyuan Avenue, Shenzhen University Town, Shenzhen, Guangdong, P.R. China, 518055.
# Correspondence may also be addressed to Guoqing Wang, Email: qing@jlu.edu.cn.

Supplementary Figure S1

Comparison of the binary classification accuracy Acc between the two algorithms McTwo and McOne. The performance is illustrated on all the 17 datasets, and each sub-figure's caption gives the dataset name. The averaged values of the classification *Sn*, *Sp*, *Acc* and *Avc* (defined as (*Sn*+*Sp*)/2) are calculated over the 30 runs of the 5-fold cross validations over the given dataset.

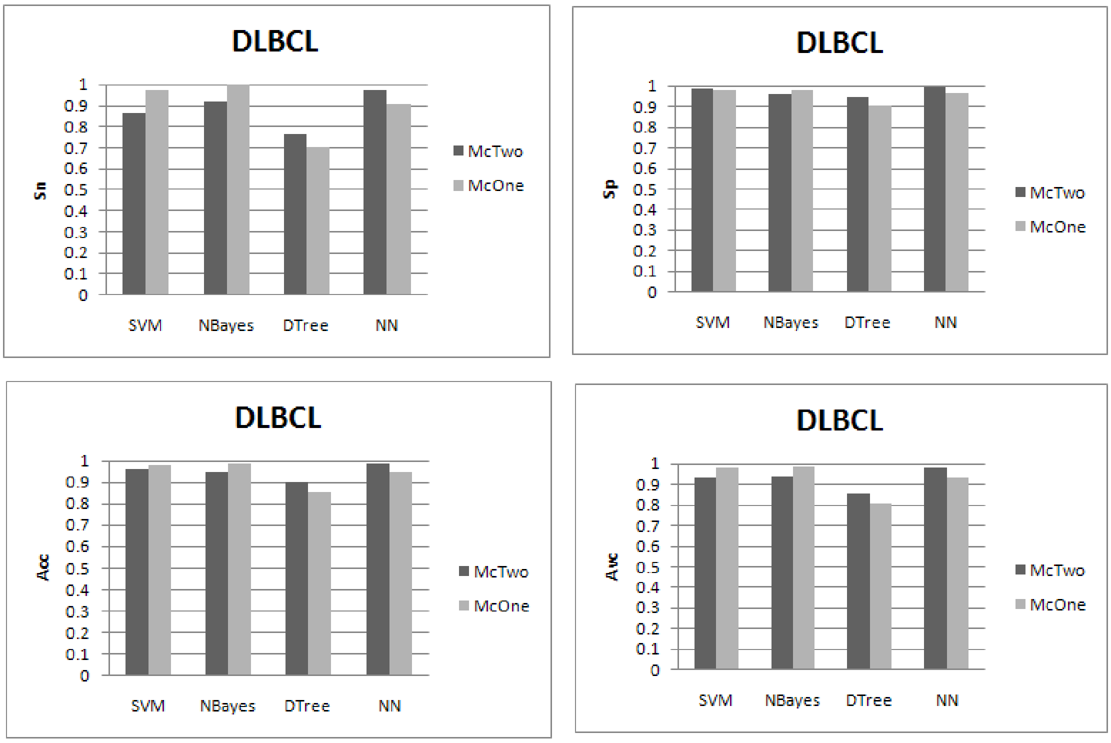

(a)

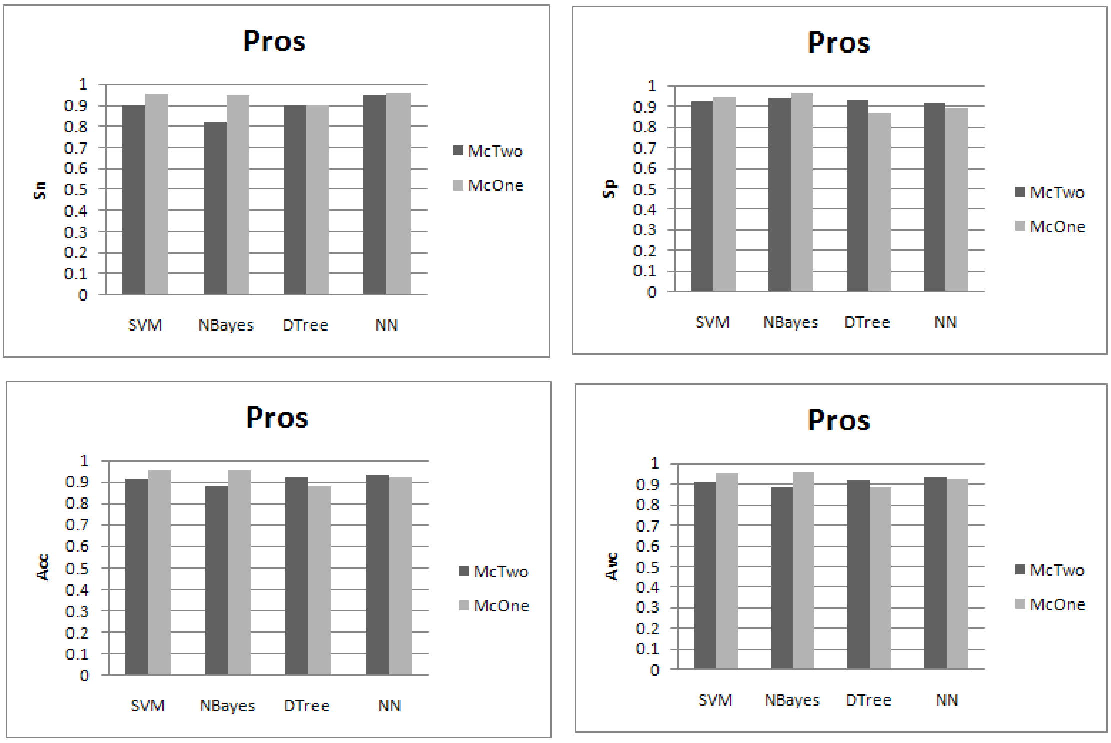

(b)

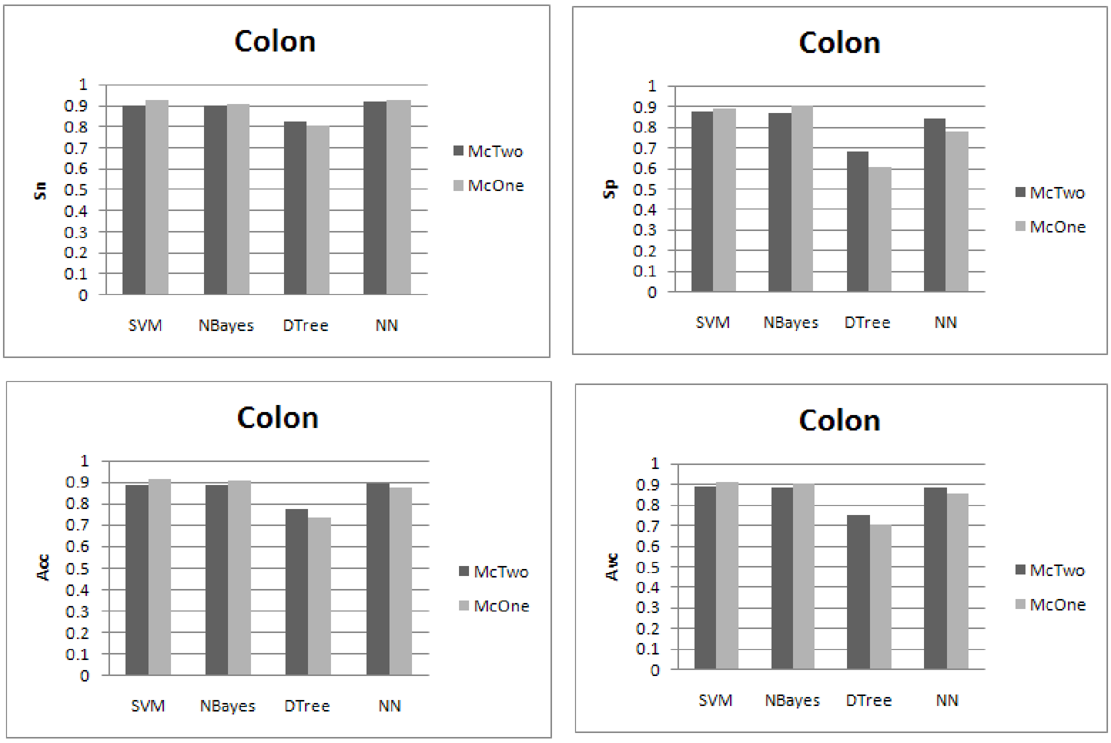
Colon
Sn
Sp
Acc
Auc
1
0.9
0.8
0.7
0.6
0.5
0.4
0.3
0.2
0.1
0
SVM
NBayes
DTree
NN
McTwo
McOne

(c)

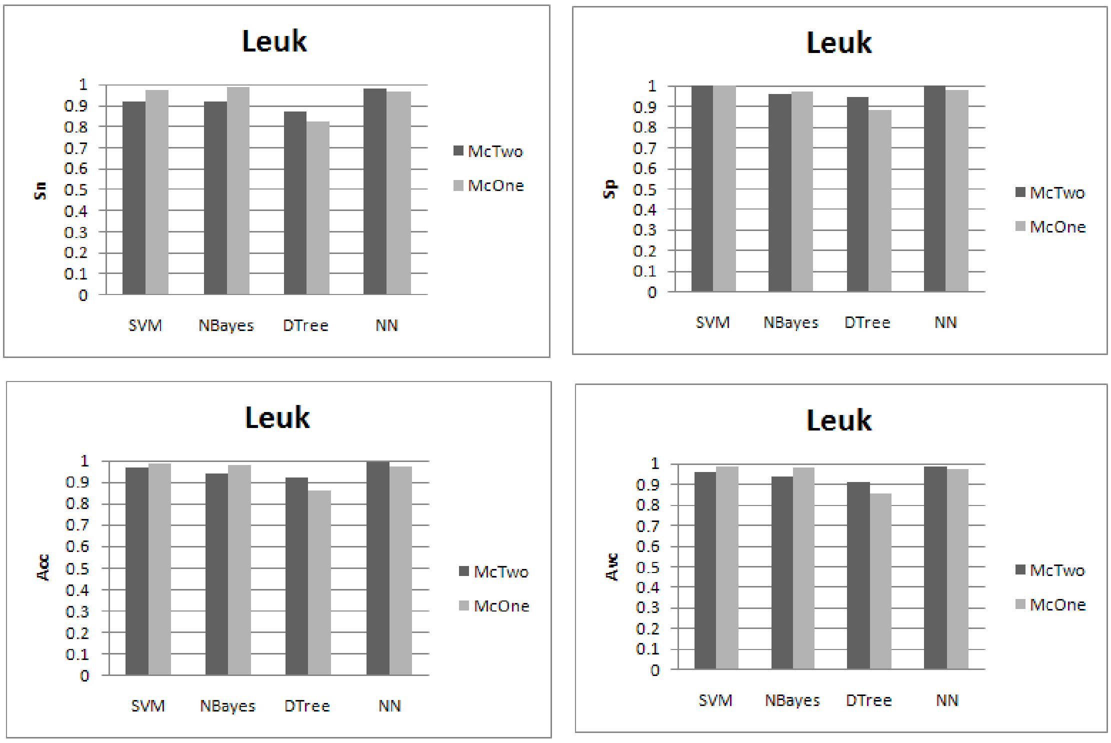
Leuk
Sn
Sp
Acc
Auc
1
0.9
0.8
0.7
0.6
0.5
0.4
0.3
0.2
0.1
0
SVM
NBayes
DTree
NN
McTwo
McOne

(d)

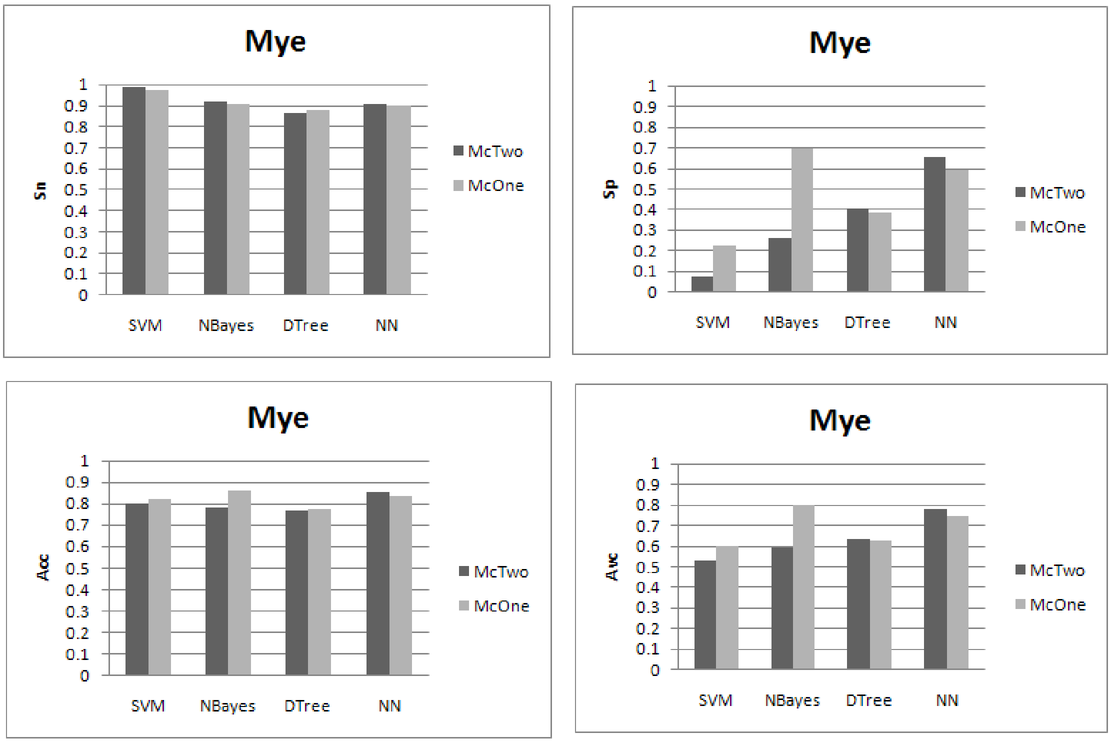
Mye
Sn
1
0.9
0.8
0.7
0.6
0.5
0.4
0.3
0.2
0.1
0
SVM
NBayes
DTree
NN
McTwo
McOne
Mye
Sp
SVM
NBayes
DTree
NN
McTwo
McOne
Mye
Acc
SVM
NBayes
DTree
NN
McTwo
McOne
Mye
Avc
SVM
NBayes
DTree
NN
McTwo
McOne

(e)

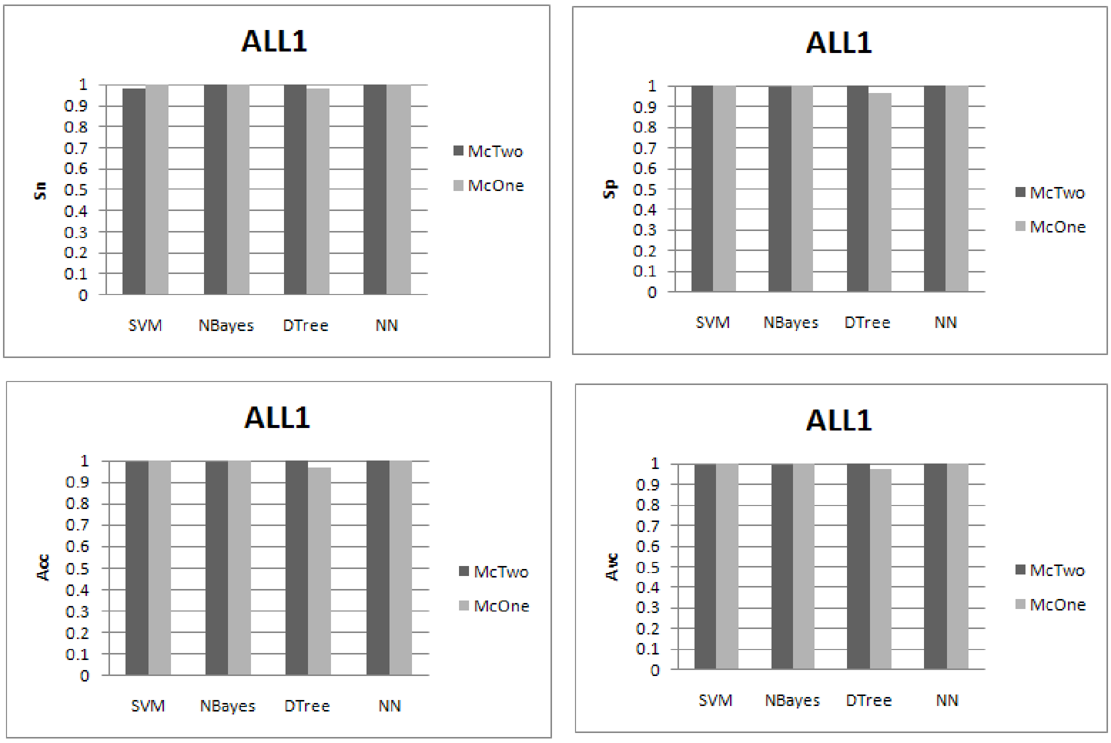
ALL1
Sn
1
0.9
0.8
0.7
0.6
0.5
0.4
0.3
0.2
0.1
0
SVM
NBayes
DTree
NN
McTwo
McOne
ALL1
Sp
SVM
NBayes
DTree
NN
McTwo
McOne
ALL1
Acc
SVM
NBayes
DTree
NN
McTwo
McOne
ALL1
Avc
SVM
NBayes
DTree
NN
McTwo
McOne

(f)

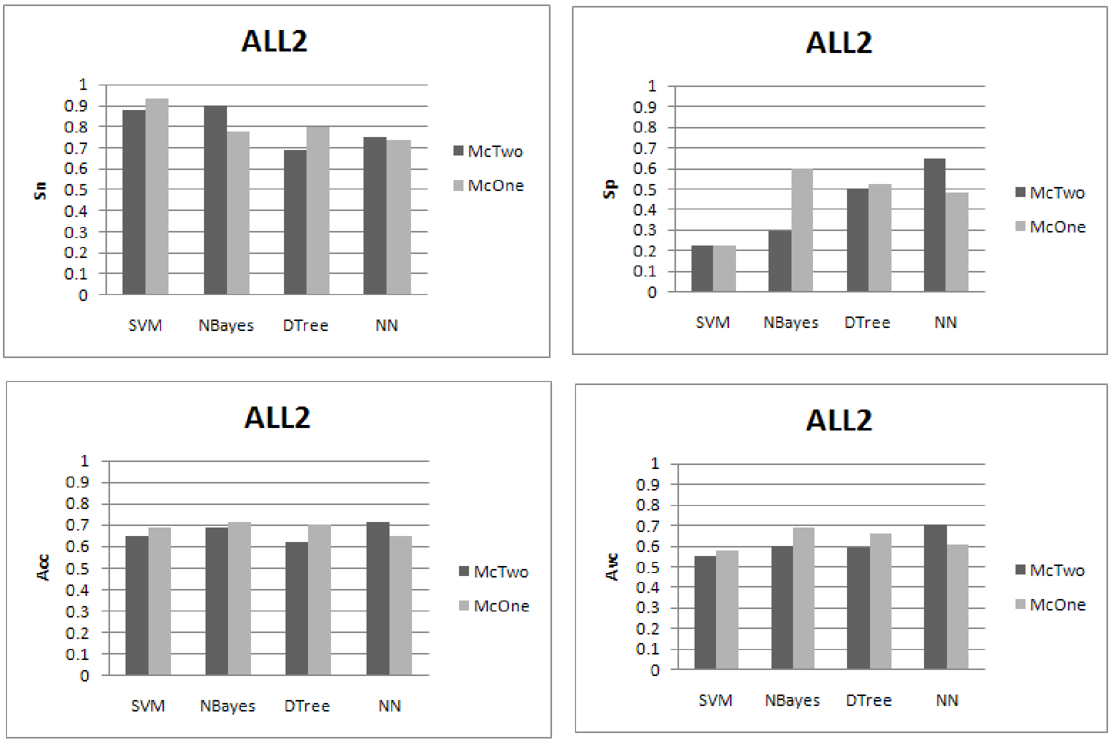
ALL2
Sn
SVM
NBayes
DTree
NN
McTwo
McOne
ALL2
Sp
SVM
NBayes
DTree
NN
McTwo
McOne
ALL2
Acc
SVM
NBayes
DTree
NN
McTwo
McOne
ALL2
Avc
SVM
NBayes
DTree
NN
McTwo
McOne

(g)

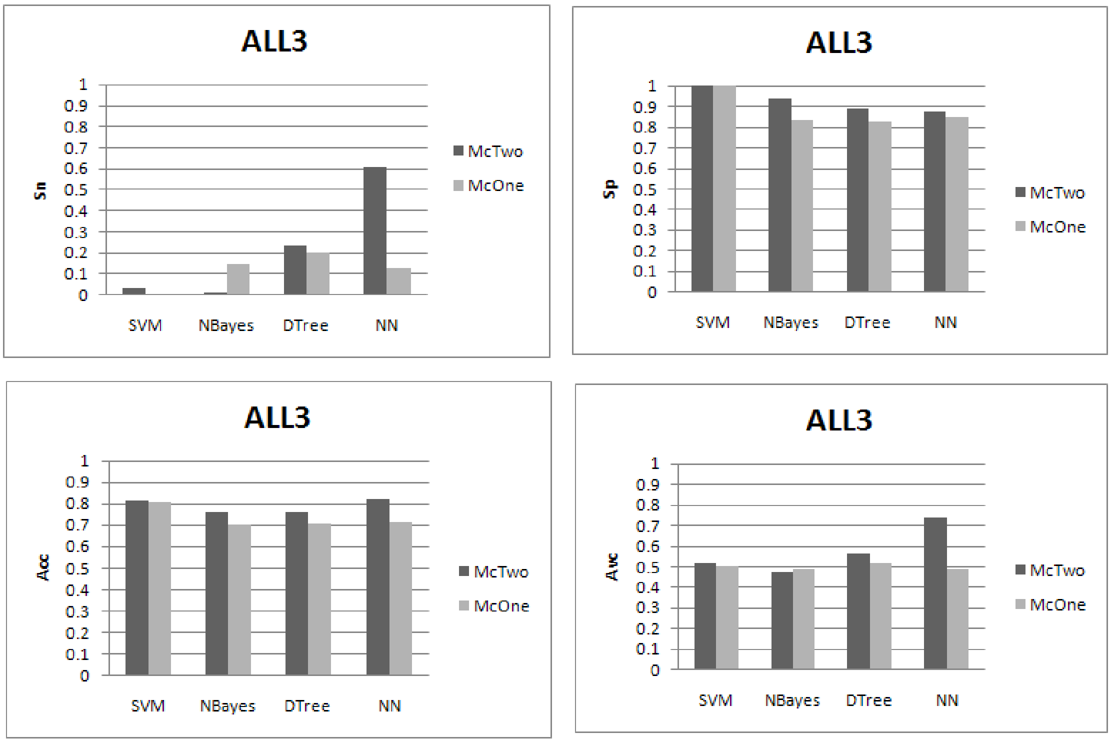
ALL3
Sn
SVM
NBayes
DTree
NN
McTwo
McOne
ALL3
Sp
SVM
NBayes
DTree
NN
McTwo
McOne
ALL3
Acc
SVM
NBayes
DTree
NN
McTwo
McOne
ALL3
Avc
SVM
NBayes
DTree
NN
McTwo
McOne

(h)

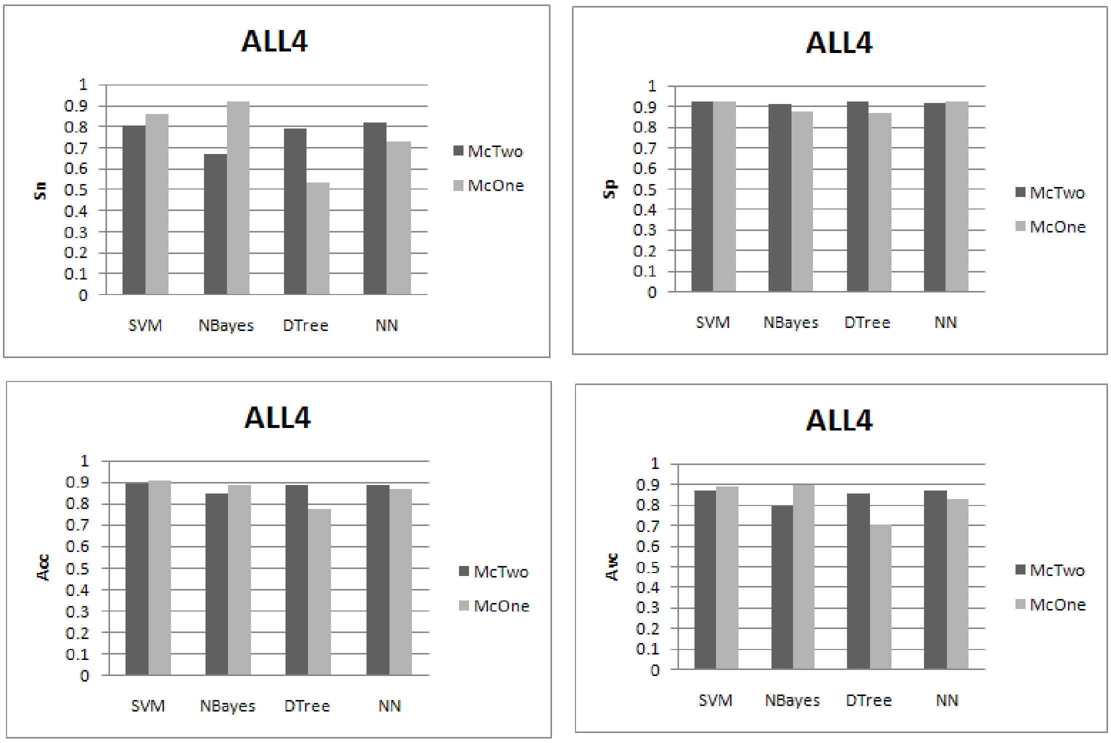

ALL4
Sn
Sp
Acc
Auc
SVM
NBayes
DTree
NN
McTwo
McOne

(i)

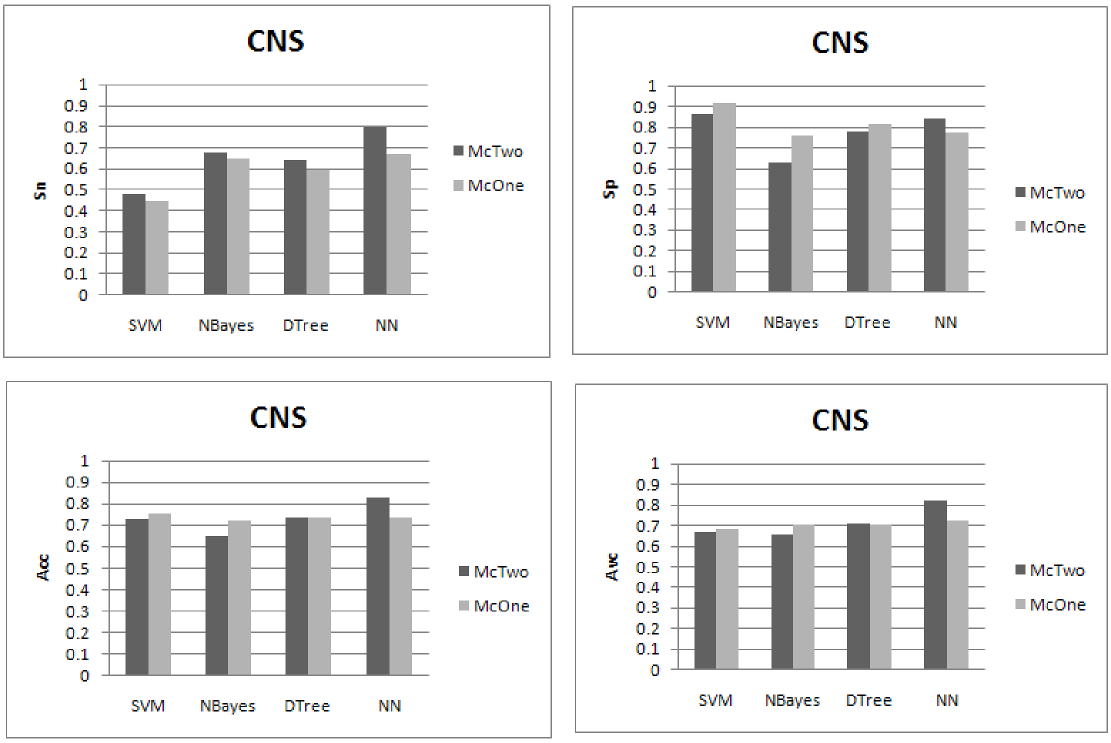

CNS
Sn
Sp
Acc
Auc
SVM
NBayes
DTree
NN
McTwo
McOne

(j)

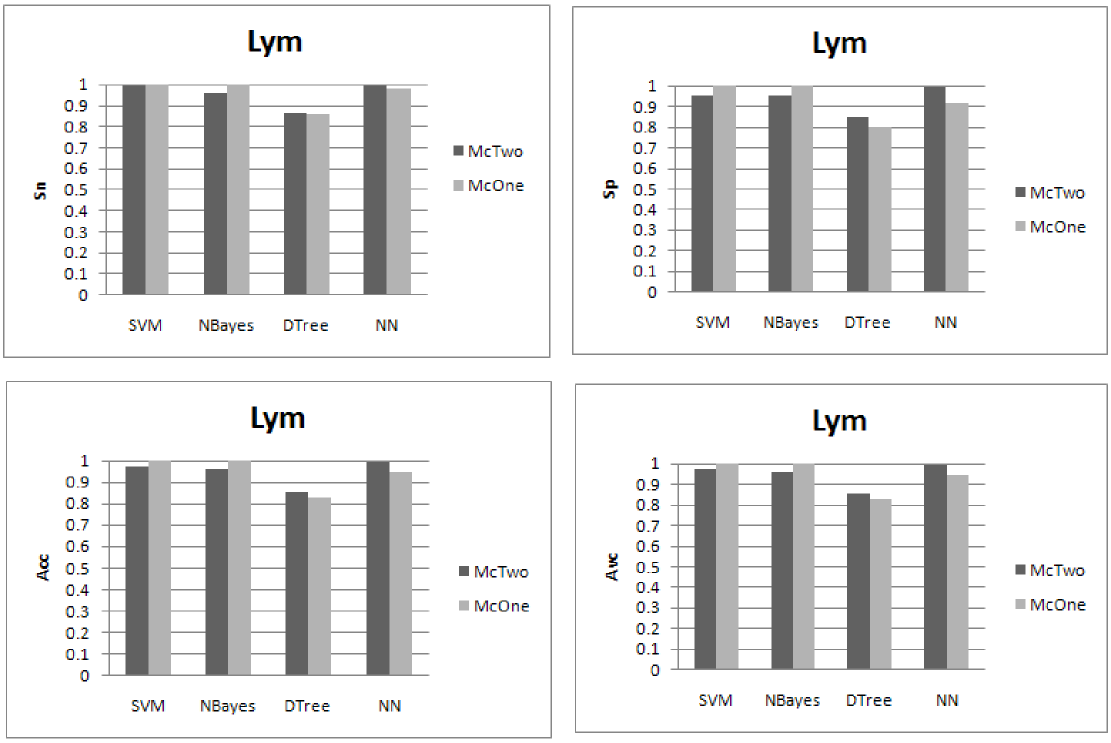
Lym
Sn
Sp
Acc
Auc
1
0.9
0.8
0.7
0.6
0.5
0.4
0.3
0.2
0.1
0
SVM
NBayes
DTree
NN
McTwo
McOne

(k)

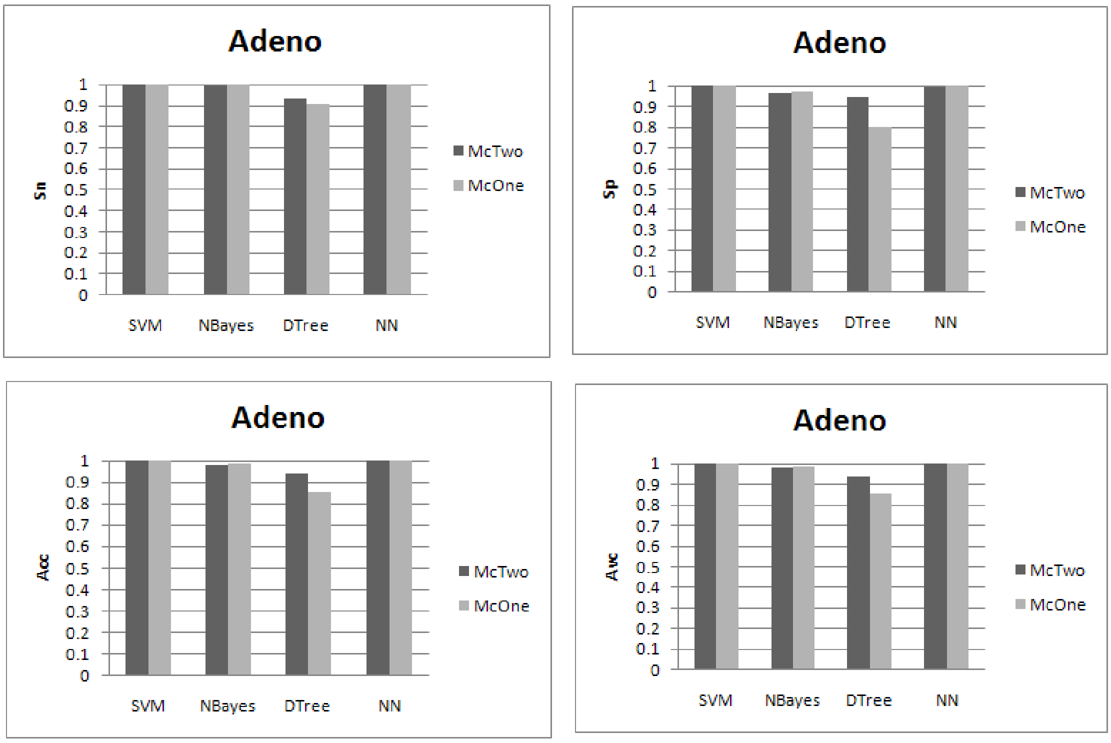
Adeno
Sn
Sp
Acc
Auc
1
0.9
0.8
0.7
0.6
0.5
0.4
0.3
0.2
0.1
0
SVM
NBayes
DTree
NN
McTwo
McOne

(l)

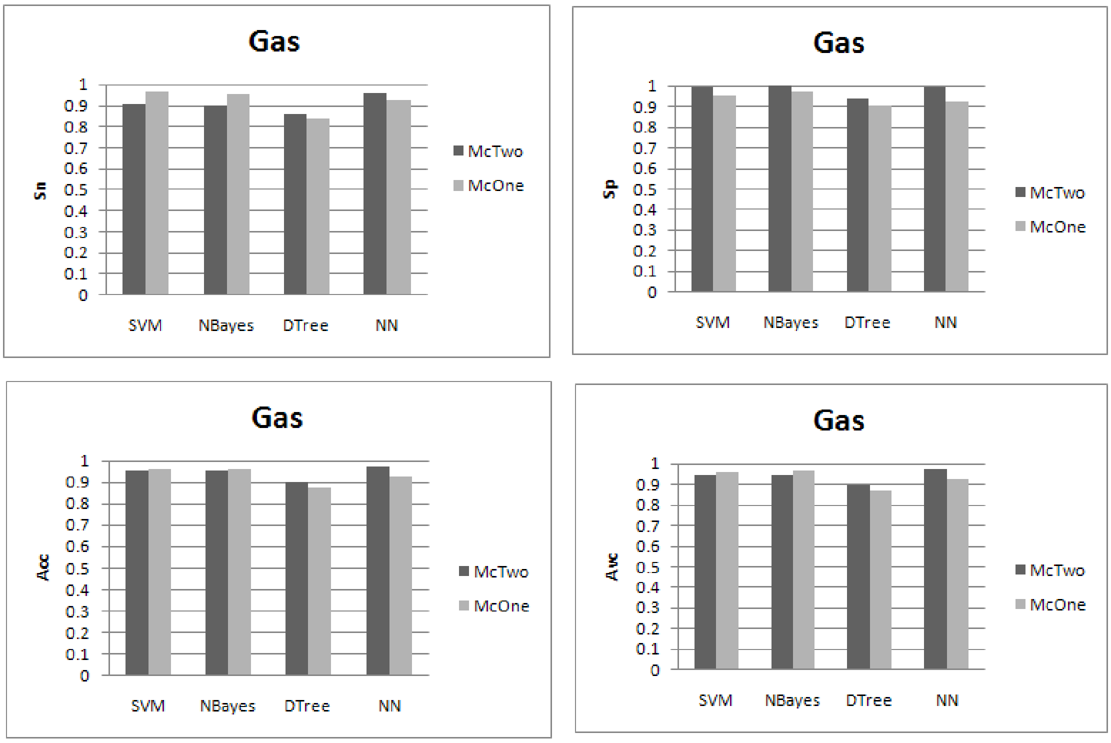
Gas
Sn
SVM
NBayes
DTree
NN
McTwo
McOne
Gas
Sp
SVM
NBayes
DTree
NN
McTwo
McOne
Gas
Acc
SVM
NBayes
DTree
NN
McTwo
McOne
Gas
Avc
SVM
NBayes
DTree
NN
McTwo
McOne

(m)

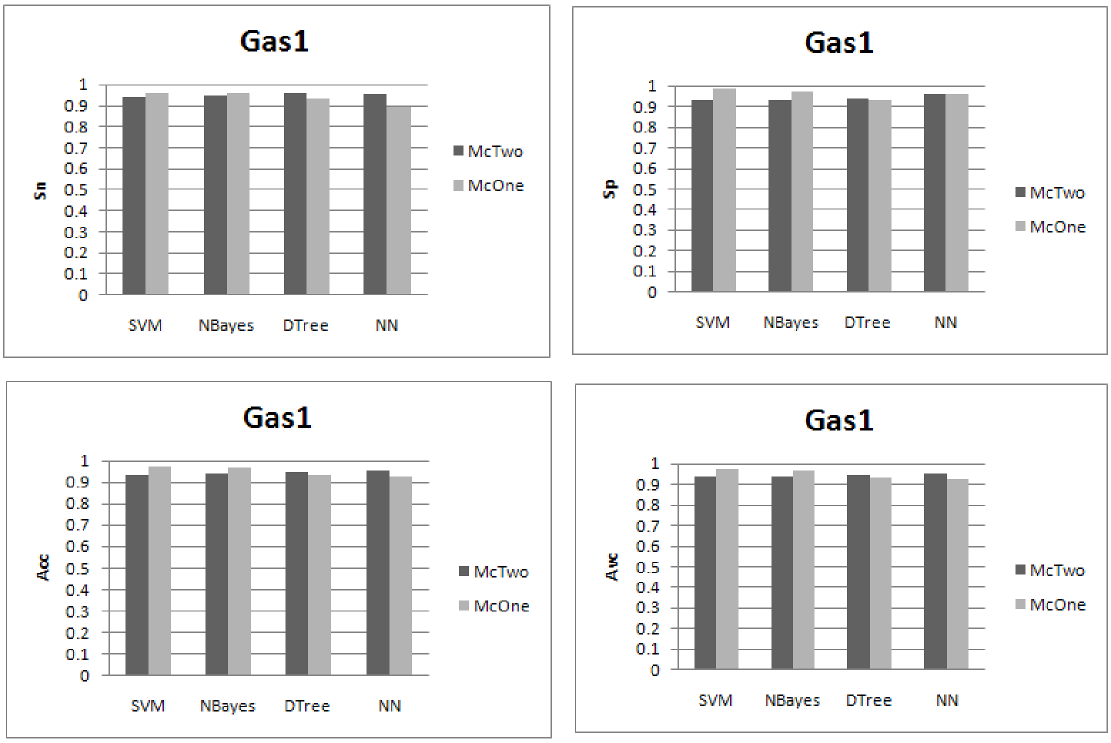
Gas1
Sn
SVM
NBayes
DTree
NN
McTwo
McOne
Gas1
Sp
SVM
NBayes
DTree
NN
McTwo
McOne
Gas1
Acc
SVM
NBayes
DTree
NN
McTwo
McOne
Gas1
Avc
SVM
NBayes
DTree
NN
McTwo
McOne

(n)

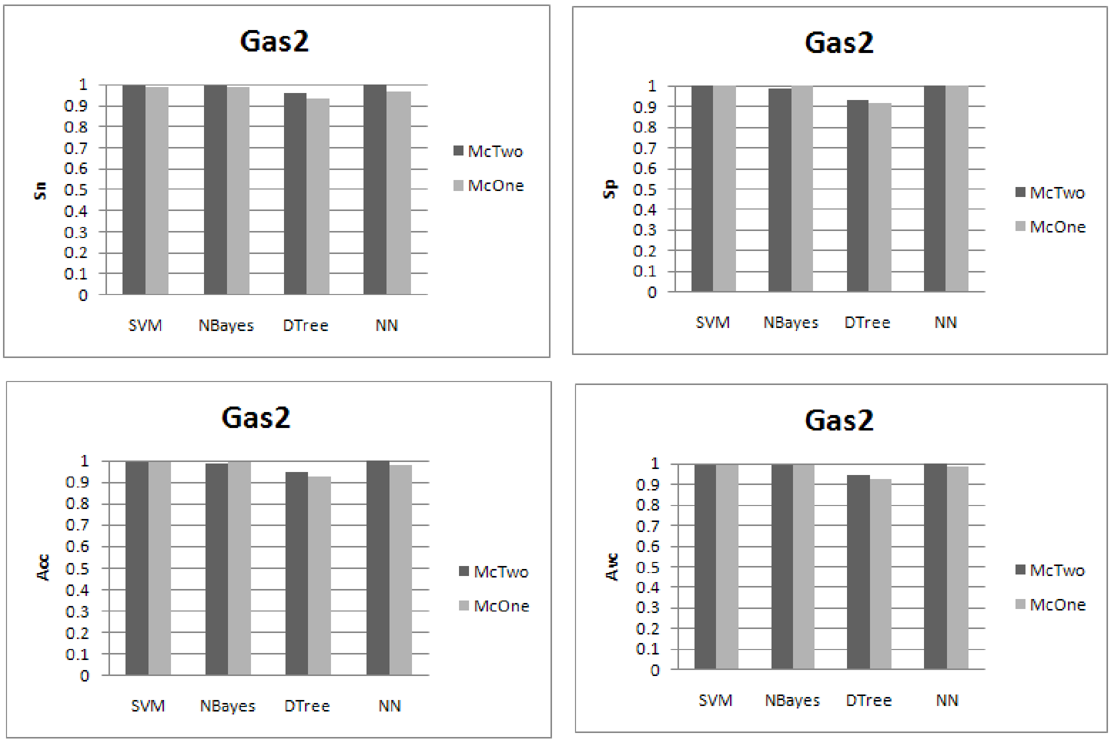
Gas2
Sn
McTwo
McOne
SVM
NBayes
DTree
NN
Gas2
Sp
McTwo
McOne
SVM
NBayes
DTree
NN
Gas2
Acc
McTwo
McOne
SVM
NBayes
DTree
NN
Gas2
Avc
McTwo
McOne
SVM
NBayes
DTree
NN
(o)

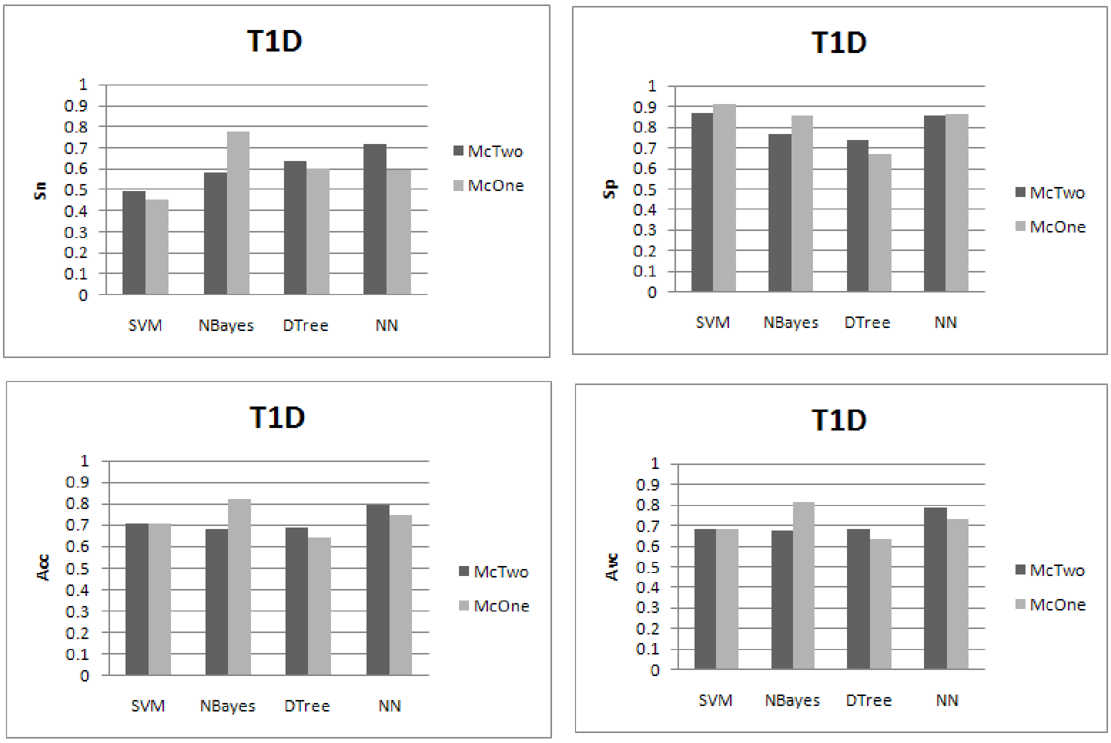
T1D
Sn
McTwo
McOne
SVM
NBayes
DTree
NN
T1D
Sp
McTwo
McOne
SVM
NBayes
DTree
NN
T1D
Acc
McTwo
McOne
SVM
NBayes
DTree
NN
T1D
Avc
McTwo
McOne
SVM
NBayes
DTree
NN
(p)

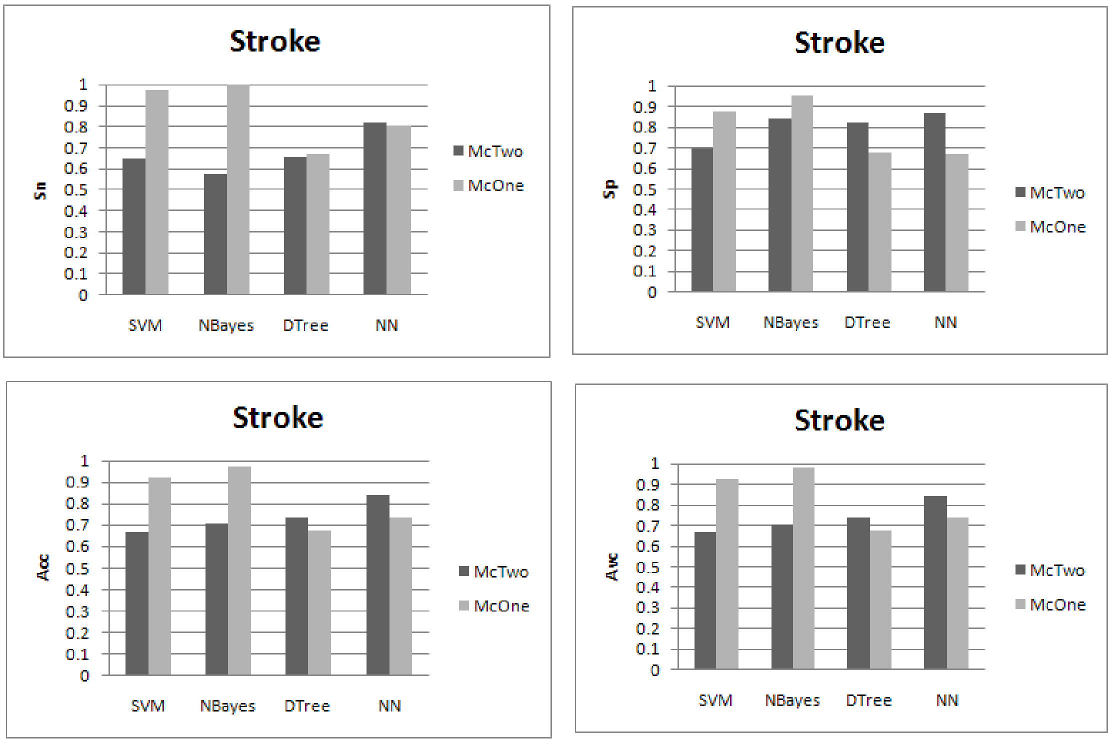
Stroke
Sn
SVM
NBayes
DTree
NN
McTwo
McOne
Stroke
Sp
SVM
NBayes
DTree
NN
McTwo
McOne
Stroke
Acc
SVM
NBayes
DTree
NN
McTwo
McOne
Stroke
Avc
SVM
NBayes
DTree
NN
McTwo
McOne

(q)

Supplementary Figure S2

Comparison of the binary classification accuracy Acc among the four algorithms, McTwo, CFS, PAM and RRF. The performance is illustrated on all the 17 datasets, and each sub-figure's caption gives the dataset name. The averaged values of the classification *Sn*, *Sp*, *Acc* and *Avc* (defined as (*Sn*+*Sp*)/2) are calculated over the 30 runs of the 5-fold cross validations over the given dataset.

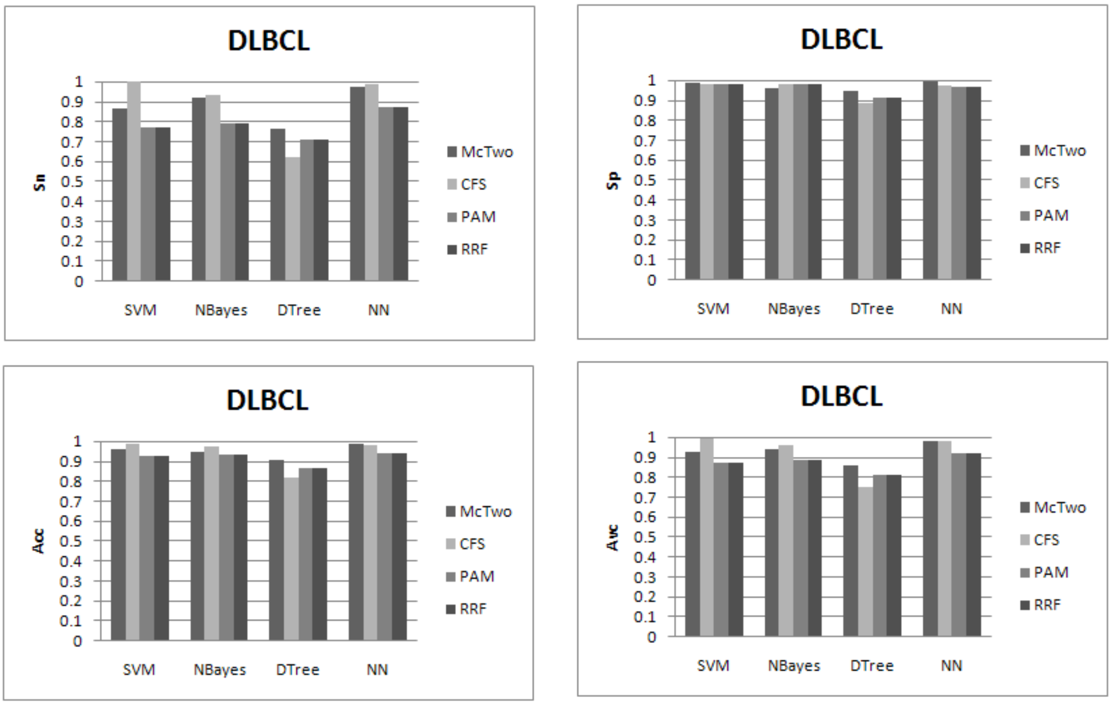

(a)

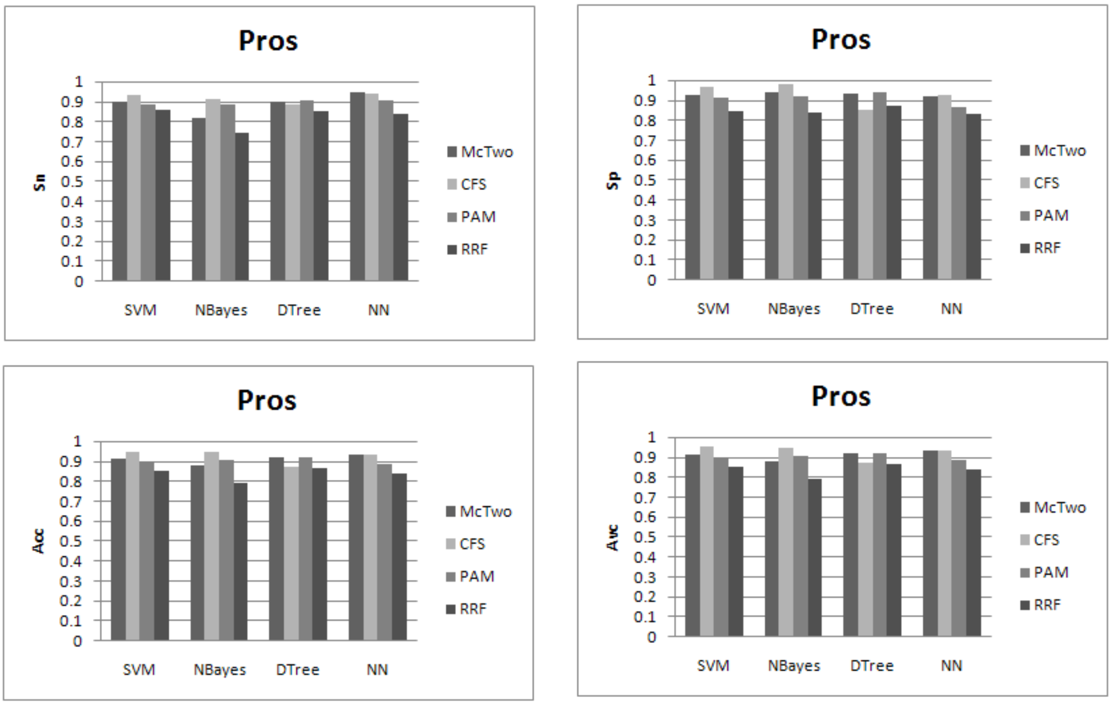

(b)

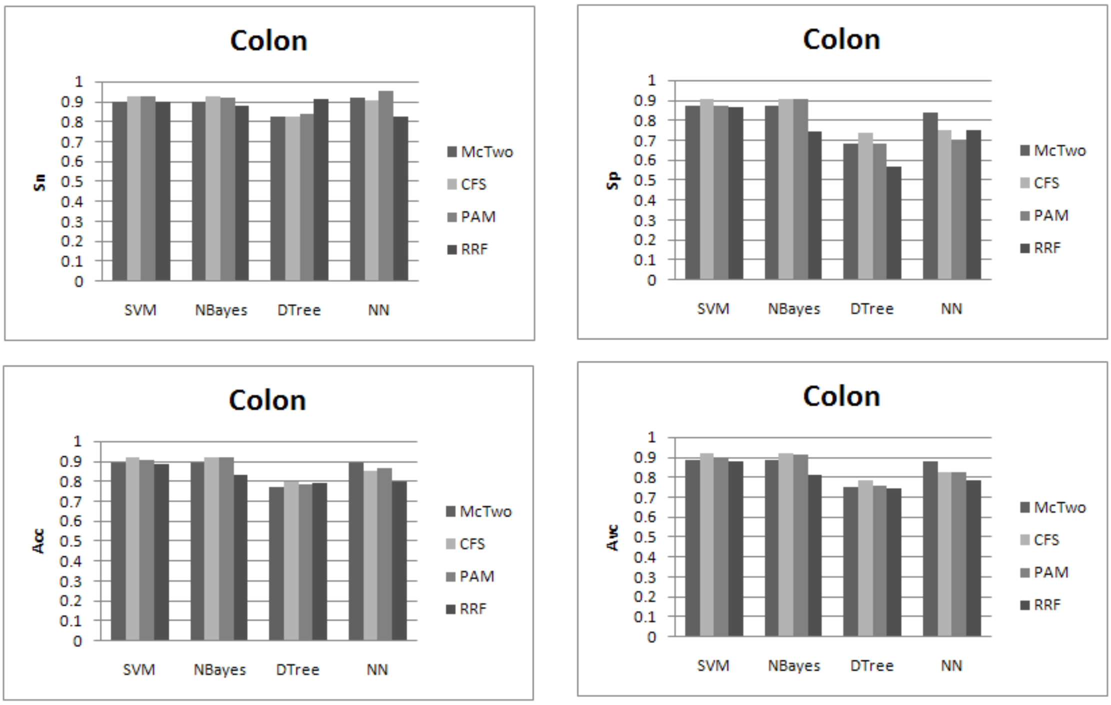
Colon
Sn
1
0.9
0.8
0.7
0.6
0.5
0.4
0.3
0.2
0.1
0
SVM
NBayes
DTree
NN
McTwo
CFS
PAM
RRF
Colon
Sp
Colon
Acc
Colon
Auc

(c)

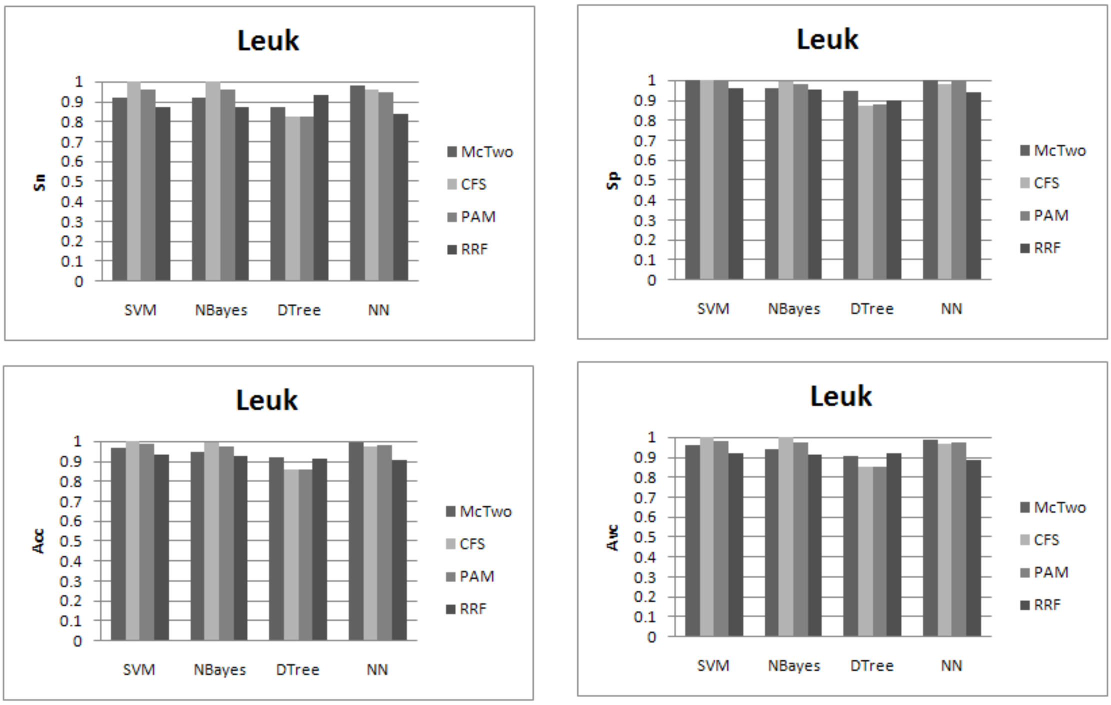
Leuk
Sn
1
0.9
0.8
0.7
0.6
0.5
0.4
0.3
0.2
0.1
0
SVM
NBayes
DTree
NN
McTwo
CFS
PAM
RRF
Leuk
Sp
Leuk
Acc
Leuk
Auc

(d)

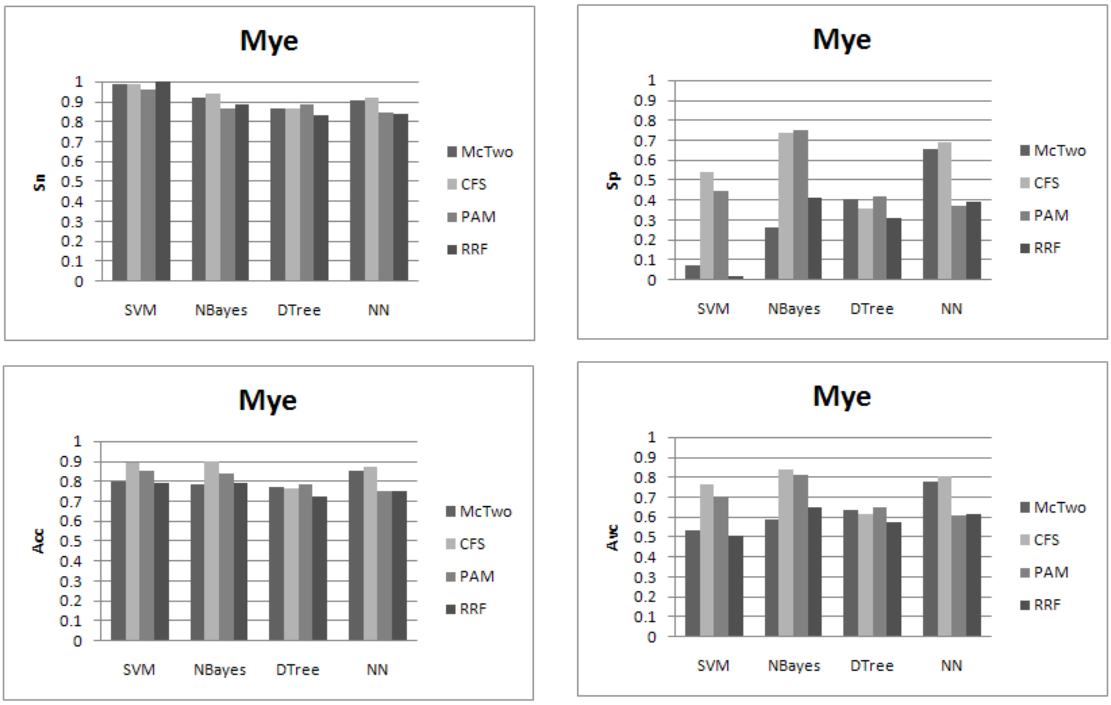
Mye
Sn
Sp
Acc
Avc
SVM
NBayes
DTree
NN
McTwo
CFS
PAM
RRF

(e)

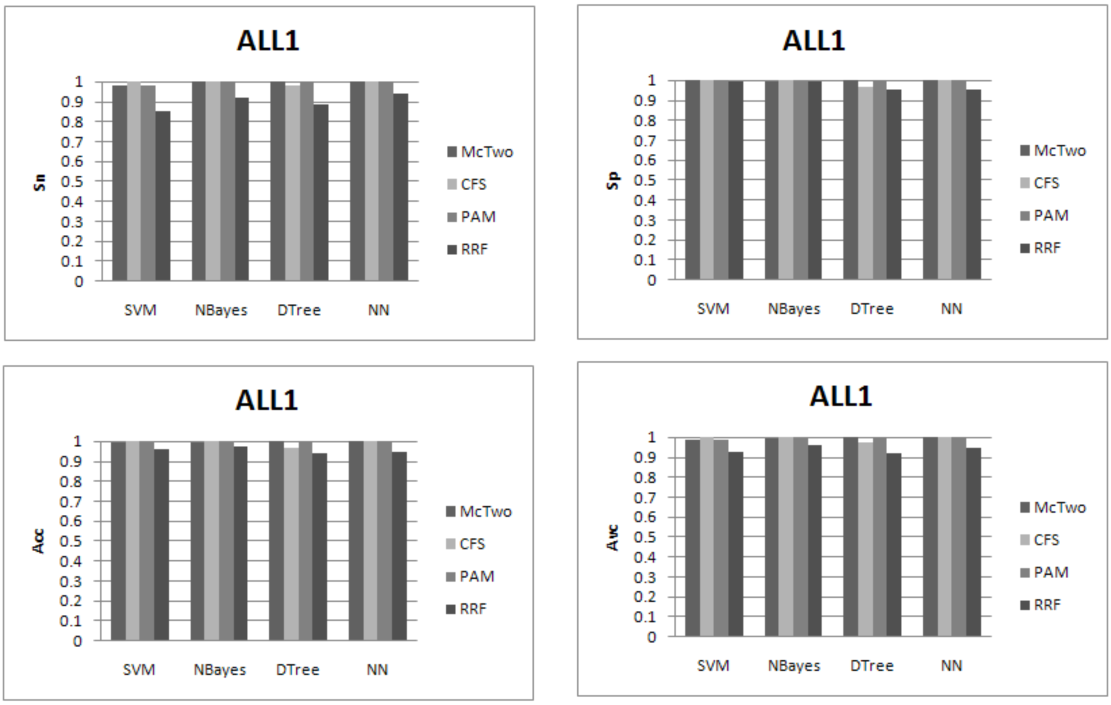
ALL1
Sn
Sp
Acc
Avc
SVM
NBayes
DTree
NN
McTwo
CFS
PAM
RRF

(f)

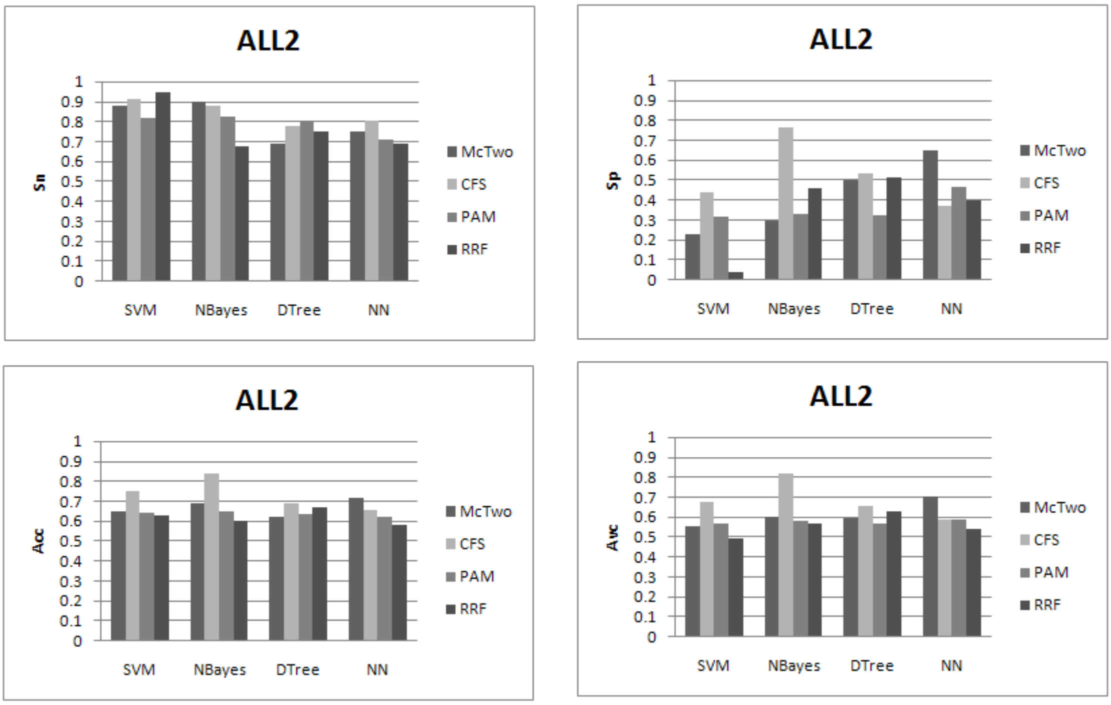
ALL2
Sn
SVM
NBayes
DTree
NN
McTwo
CFS
PAM
RRF
ALL2
Sp
ALL2
Acc
ALL2
Avc

(g)

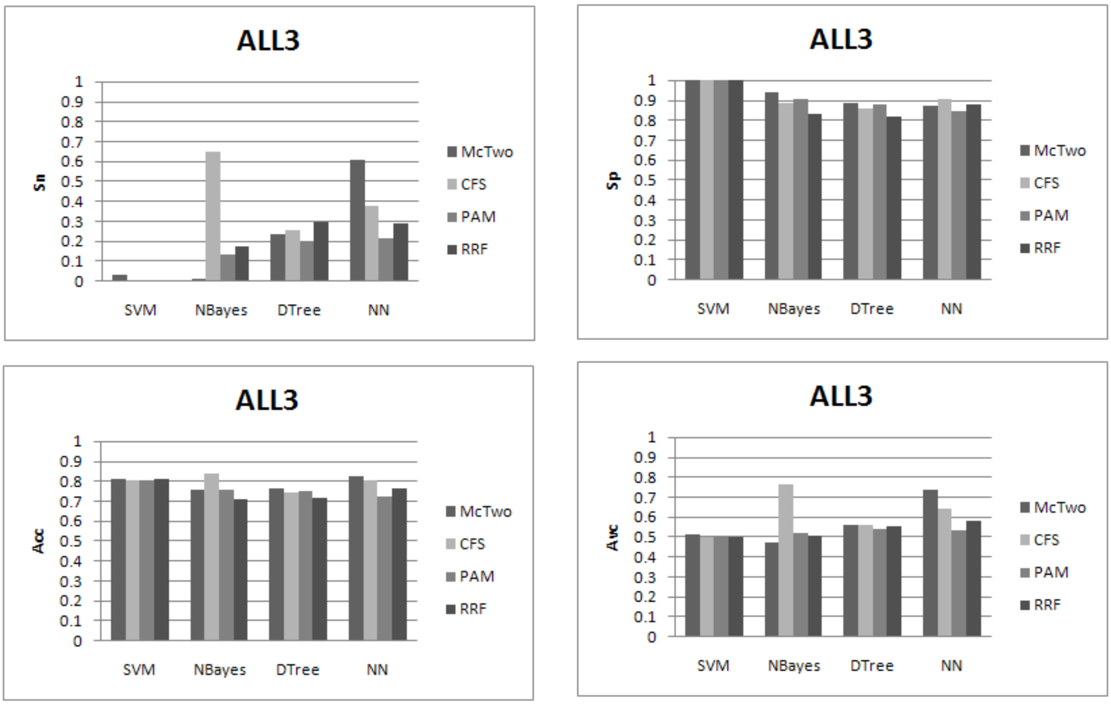
ALL3
Sn
SVM
NBayes
DTree
NN
McTwo
CFS
PAM
RRF
ALL3
Sp
ALL3
Acc
ALL3
Avc

(h)

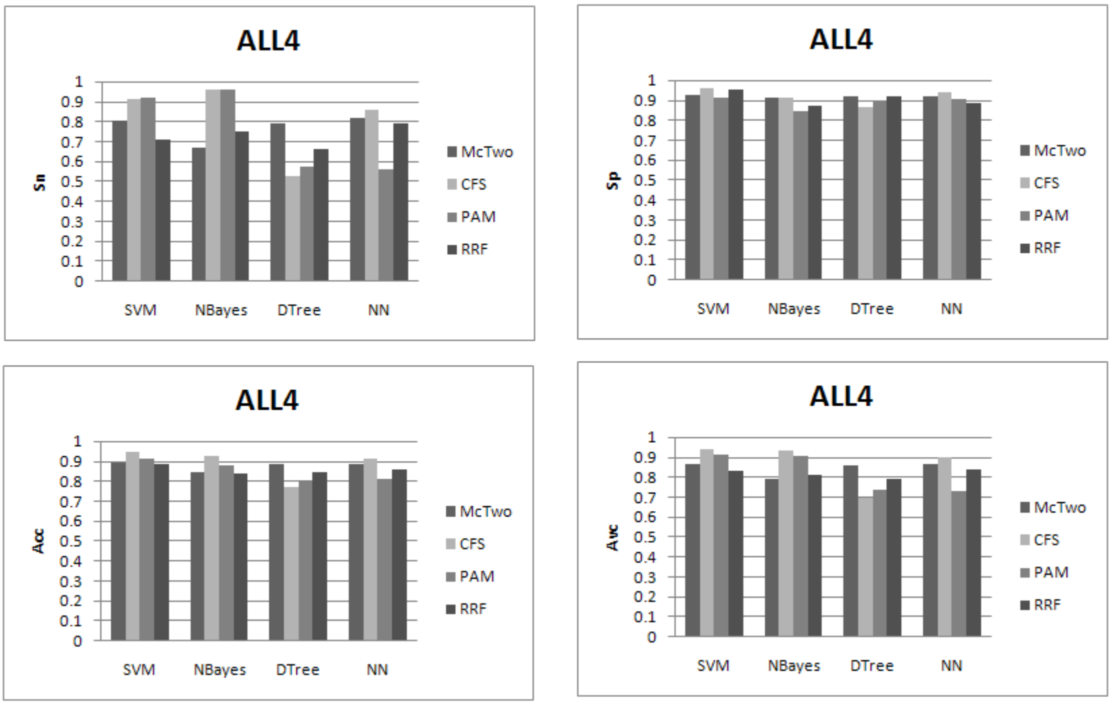
ALL4
Sn
Sp
Acc
Avc
SVM
NBayes
DTree
NN
McTwo
CFS
PAM
RRF
0
0.1
0.2
0.3
0.4
0.5
0.6
0.7
0.8
0.9
1

(i)

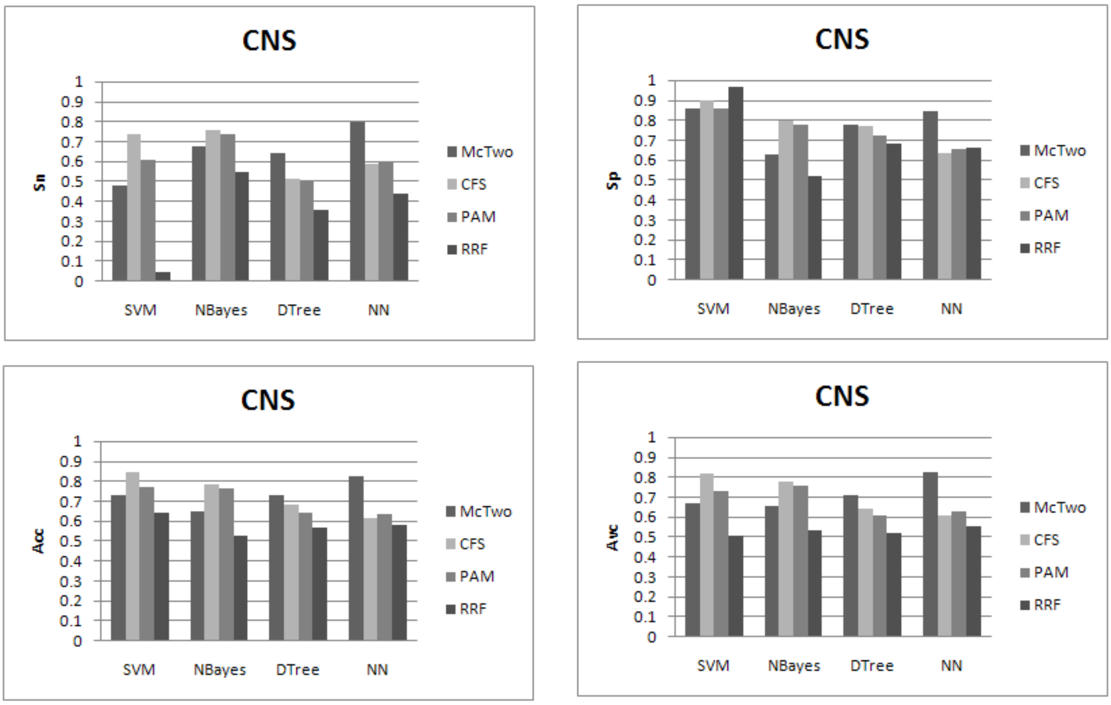
CNS
Sn
Sp
Acc
Avc
SVM
NBayes
DTree
NN
McTwo
CFS
PAM
RRF
0
0.1
0.2
0.3
0.4
0.5
0.6
0.7
0.8
0.9
1

(j)

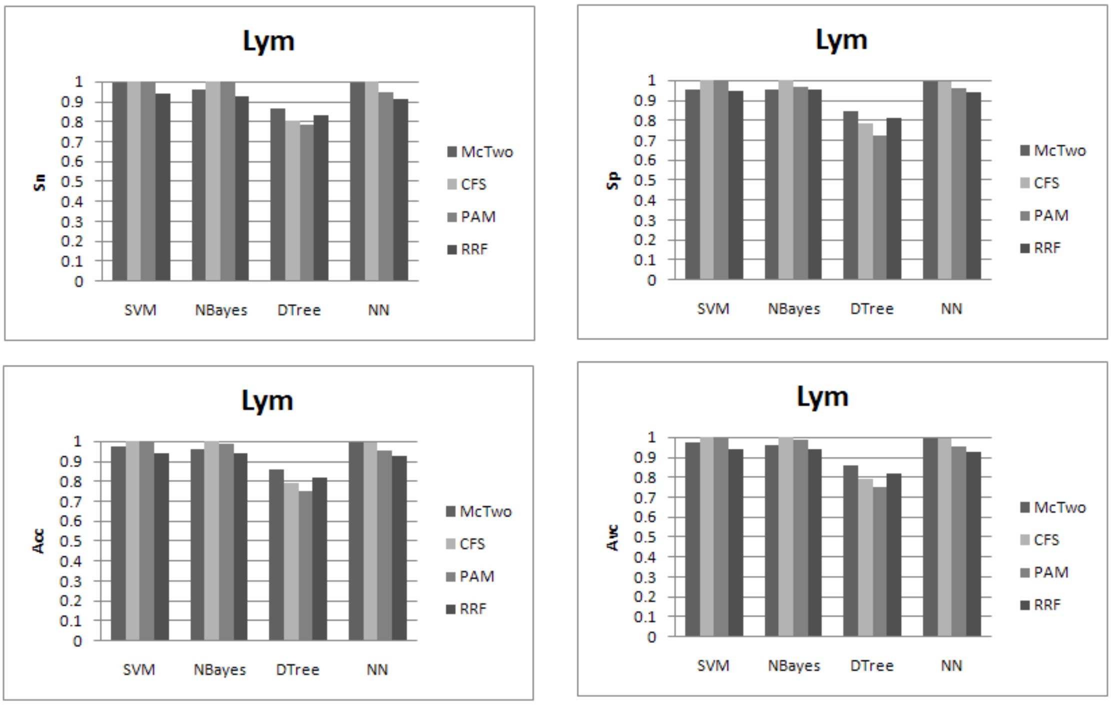
Lym
Sn
1
0.9
0.8
0.7
0.6
0.5
0.4
0.3
0.2
0.1
0
SVM
NBayes
DTree
NN
McTwo
CFS
PAM
RRF
Lym
Sp
SVM
NBayes
DTree
NN
McTwo
CFS
PAM
RRF
Lym
Acc
SVM
NBayes
DTree
NN
McTwo
CFS
PAM
RRF
Lym
Avc
SVM
NBayes
DTree
NN
McTwo
CFS
PAM
RRF

(k)

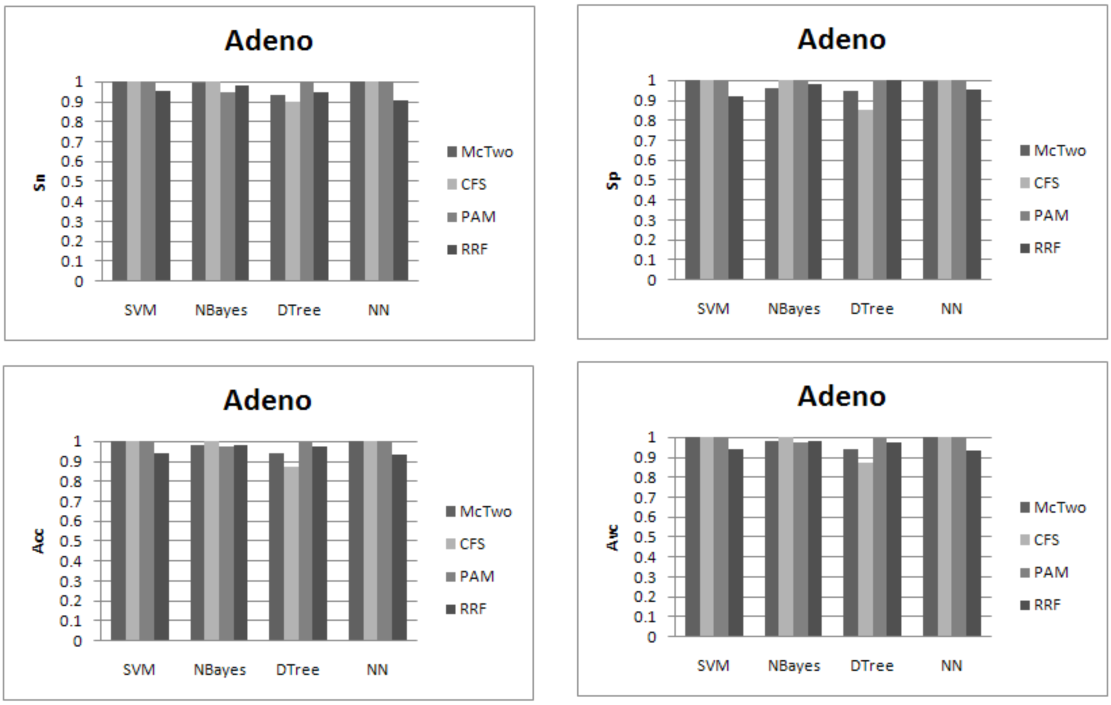
Adeno
Sn
1
0.9
0.8
0.7
0.6
0.5
0.4
0.3
0.2
0.1
0
SVM
NBayes
DTree
NN
McTwo
CFS
PAM
RRF
Adeno
Sp
SVM
NBayes
DTree
NN
McTwo
CFS
PAM
RRF
Adeno
Acc
SVM
NBayes
DTree
NN
McTwo
CFS
PAM
RRF
Adeno
Avc
SVM
NBayes
DTree
NN
McTwo
CFS
PAM
RRF

(l)

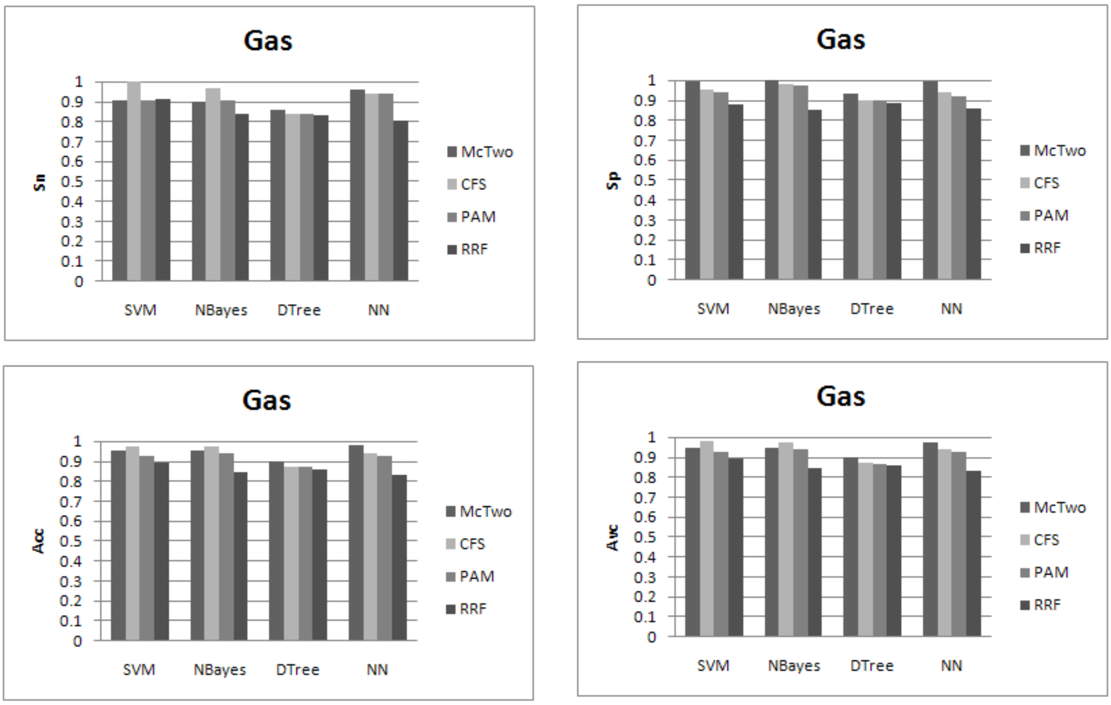
Gas
Sn
Sp
Acc
Avc
1
0.9
0.8
0.7
0.6
0.5
0.4
0.3
0.2
0.1
0
SVM
NBayes
DTree
NN
McTwo
CFS
PAM
RRF

(m)

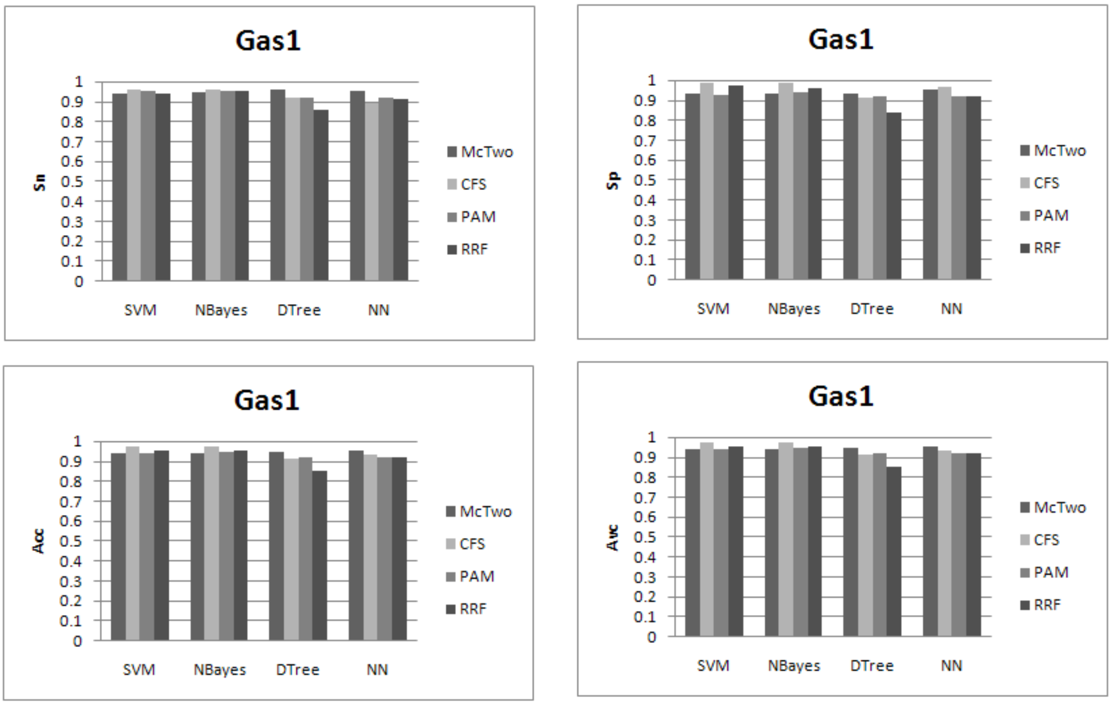
Gas1
Sn
Sp
Acc
Avc
1
0.9
0.8
0.7
0.6
0.5
0.4
0.3
0.2
0.1
0
SVM
NBayes
DTree
NN
McTwo
CFS
PAM
RRF

(n)

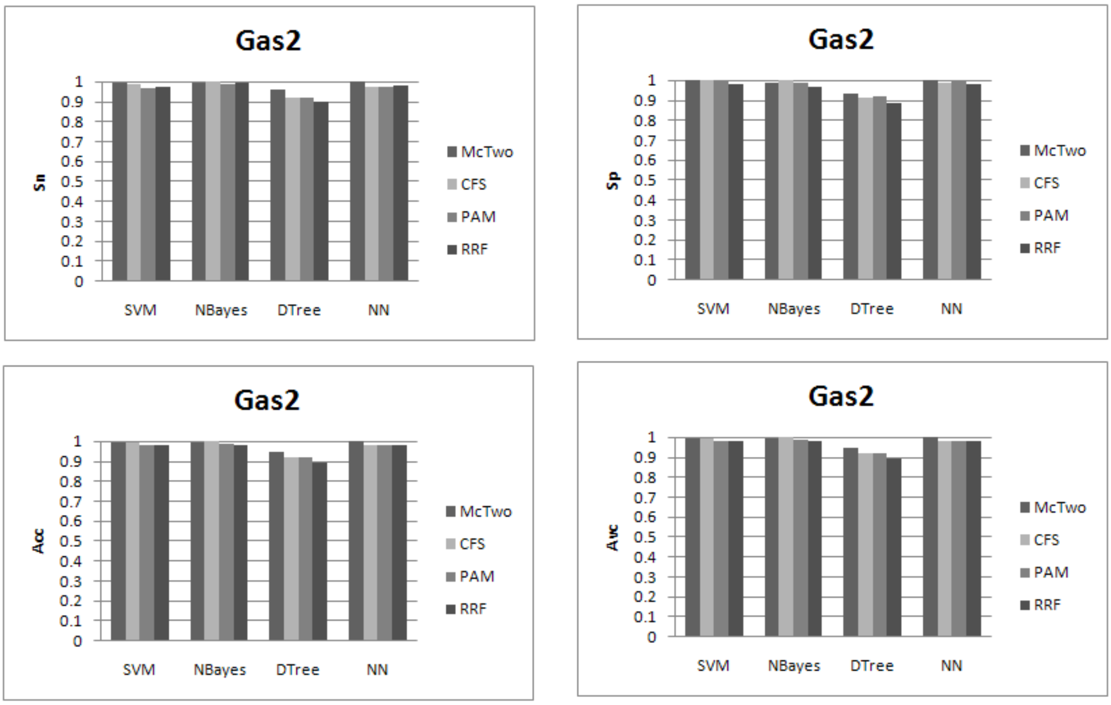
Gas2
Sn
Sp
Acc
Avc
1
0.9
0.8
0.7
0.6
0.5
0.4
0.3
0.2
0.1
0
SVM
NBayes
DTree
NN
McTwo
CFS
PAM
RRF

(o)

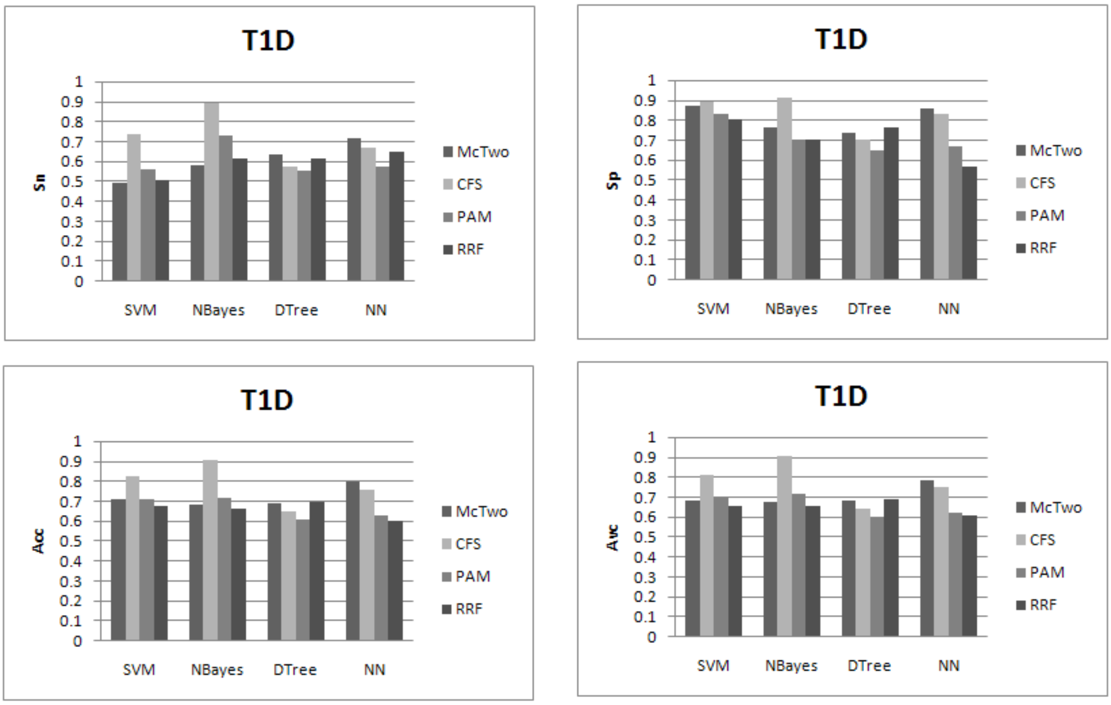
T1D
Sn
Sp
Acc
Avc
1
0.9
0.8
0.7
0.6
0.5
0.4
0.3
0.2
0.1
0
SVM
NBayes
DTree
NN
McTwo
CFS
PAM
RRF

(p)

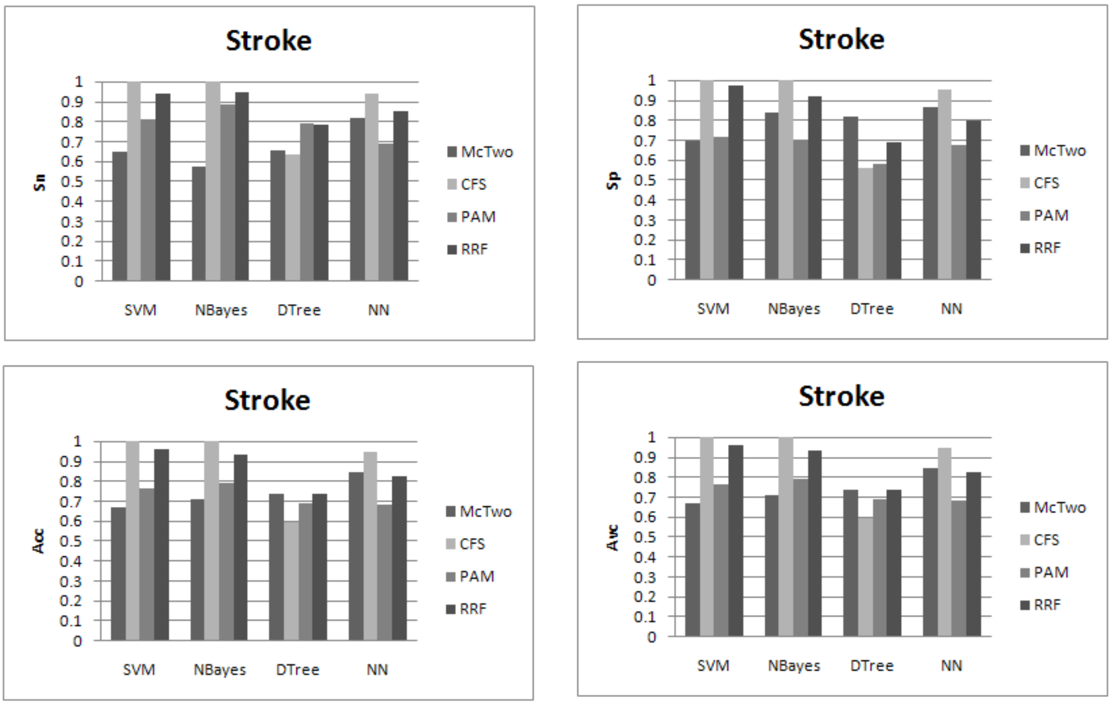

(q)

Supplementary Figure S3

Comparison of the binary classification accuracy Acc among the four algorithms, McTwo, TRank, WRank and RCORank. The performance is illustrated on all the 17 datasets, and each sub-figure's caption gives the dataset name. The averaged values of the classification *Sn*, *Sp*, *Acc* and *Avc* (defined as (*Sn*+*Sp*)/2) are calculated over the 30 runs of the 5-fold cross validations over the given dataset.

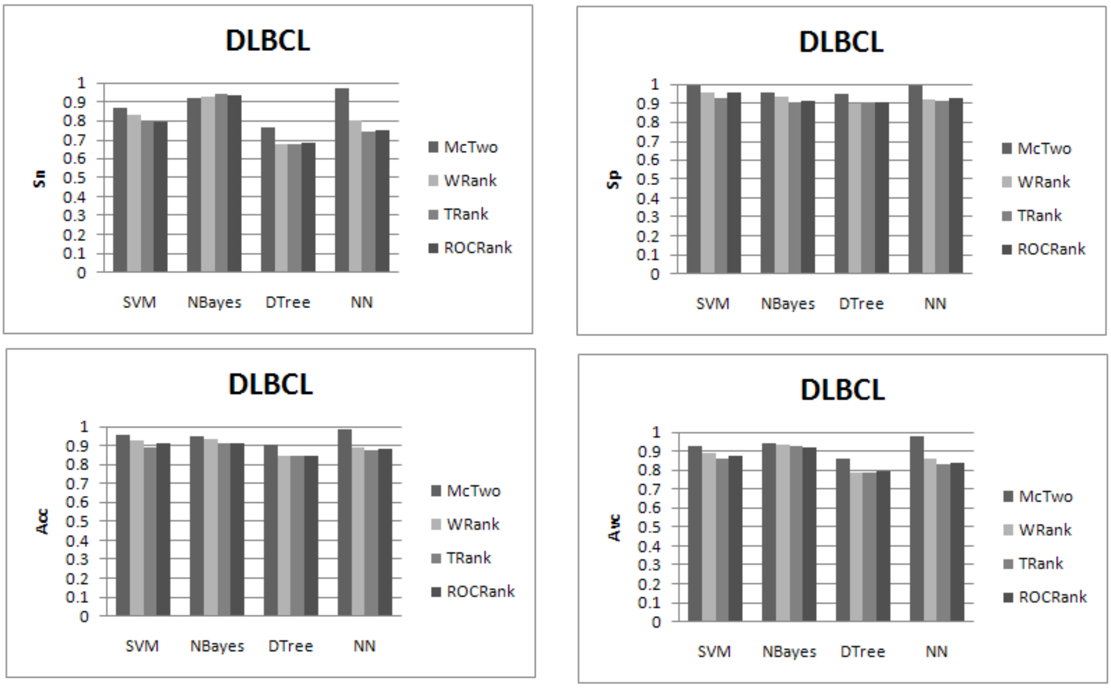

(a)

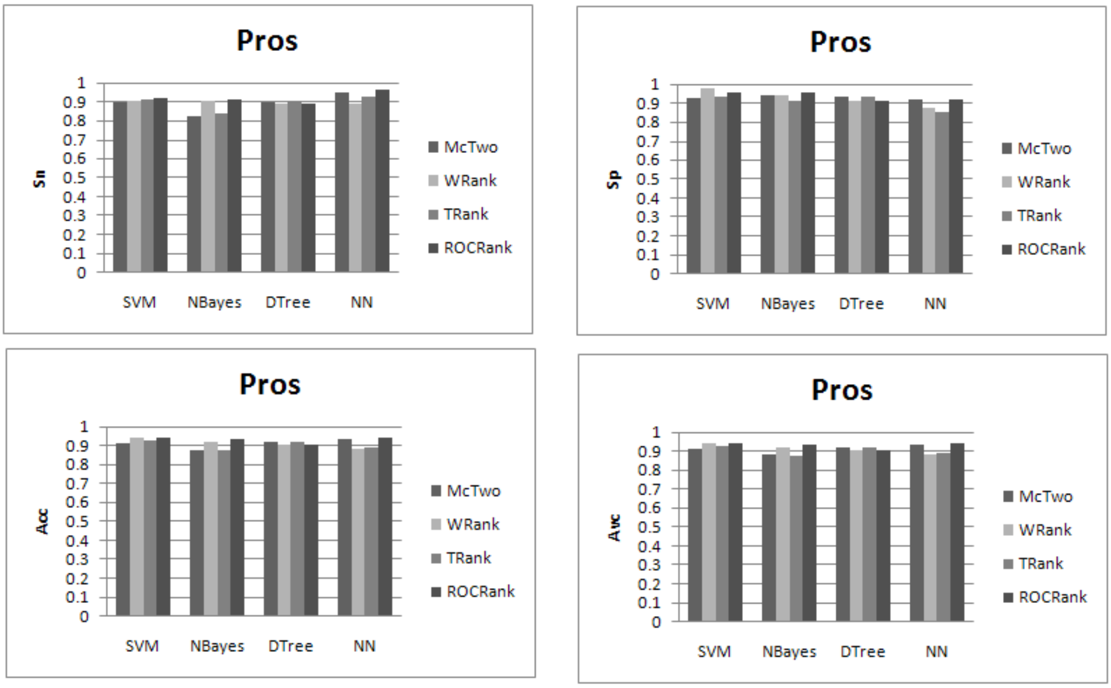

(b)

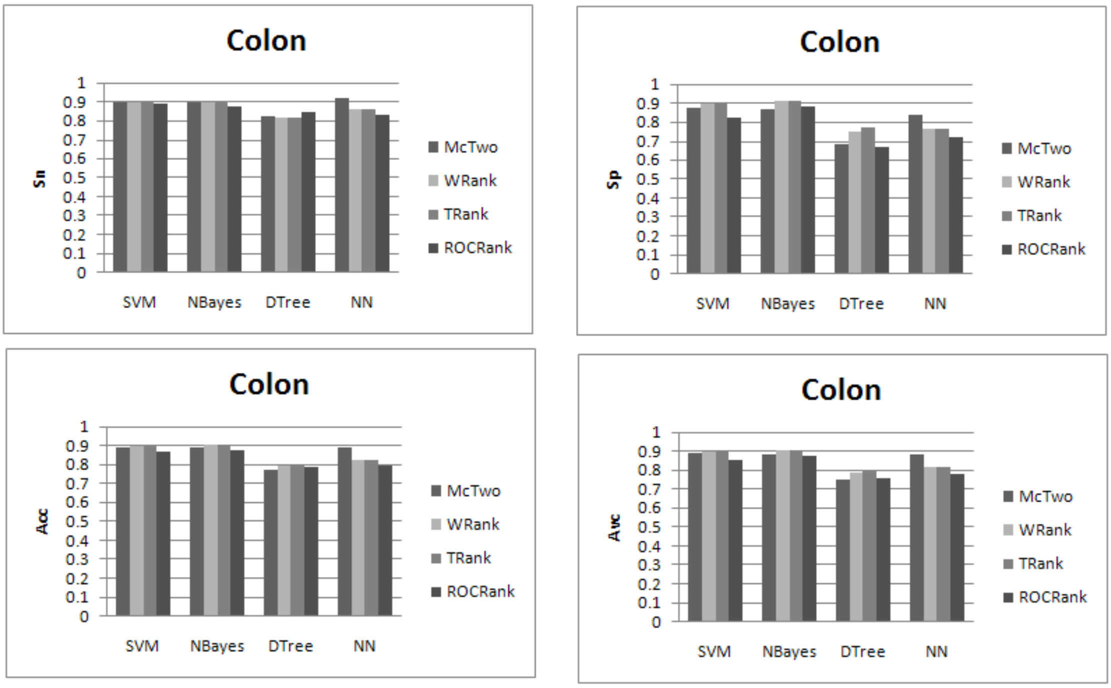
Colon
Sn
Sp
Acc
Auc
1
0.9
0.8
0.7
0.6
0.5
0.4
0.3
0.2
0.1
0
SVM
NBayes
DTree
NN
McTwo
WRank
TRank
ROCRank

(c)

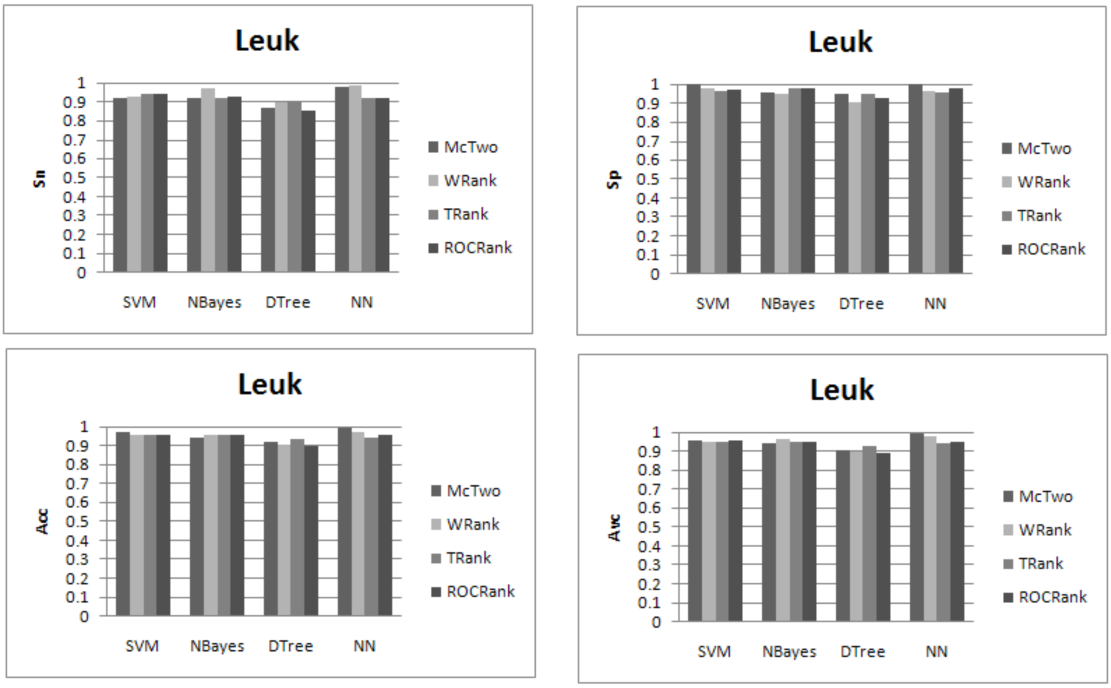
Leuk
Sn
Sp
Acc
Auc
1
0.9
0.8
0.7
0.6
0.5
0.4
0.3
0.2
0.1
0
SVM
NBayes
DTree
NN
McTwo
WRank
TRank
ROCRank

(d)

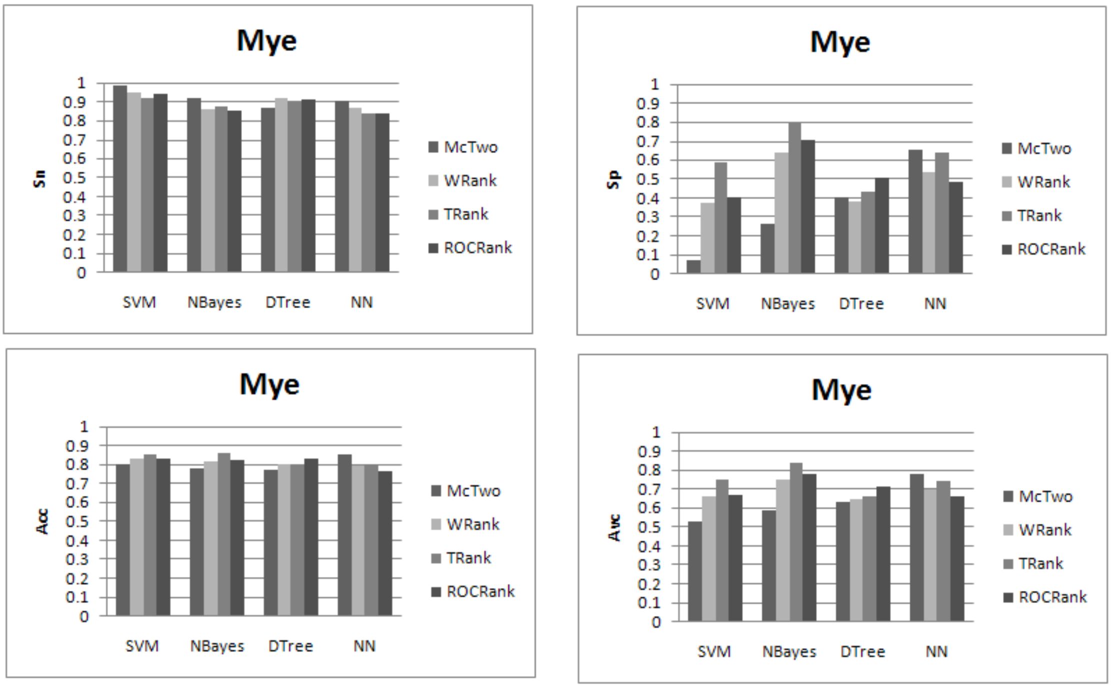
Mye
Sn
Sp
Acc
Auc
McTwo
WRank
TRank
ROCRank
SVM
NBayes
DTree
NN

(e)

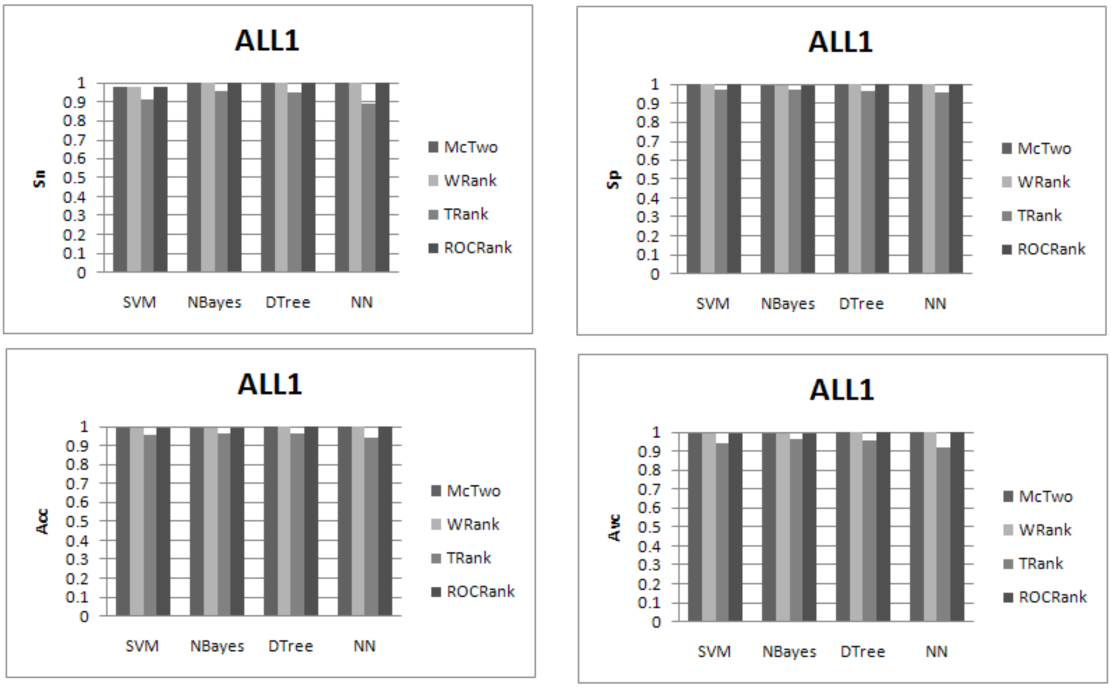
ALL1
Sn
Sp
Acc
Auc
McTwo
WRank
TRank
ROCRank
SVM
NBayes
DTree
NN

(f)

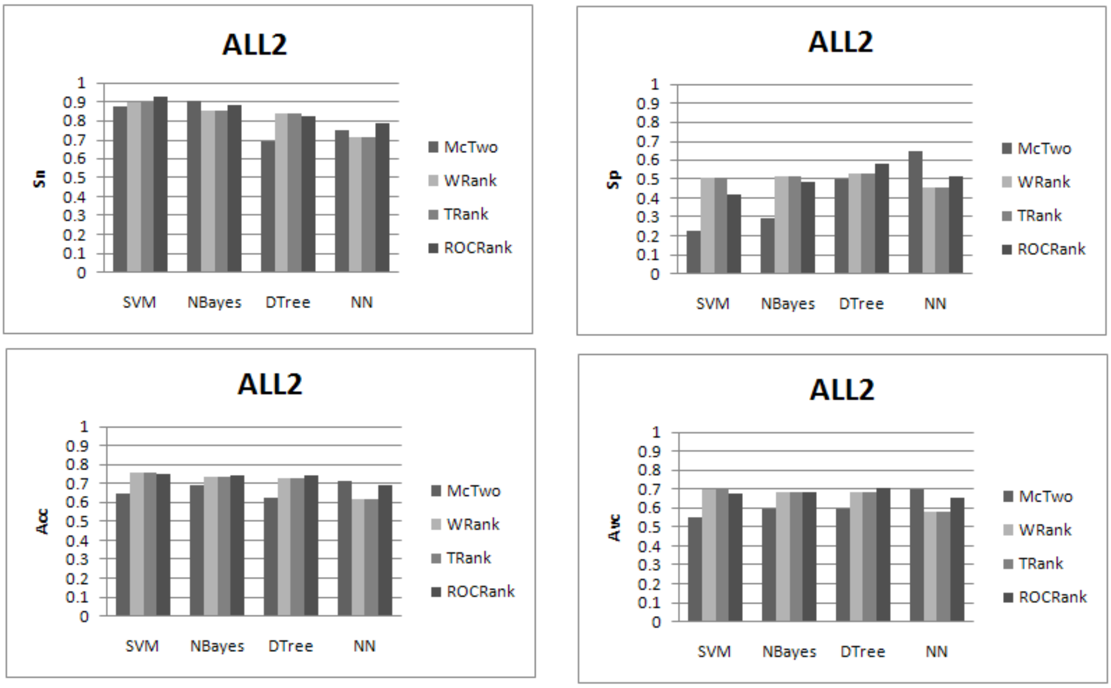
ALL2
Sn
Sp
Acc
Auc
1
0.9
0.8
0.7
0.6
0.5
0.4
0.3
0.2
0.1
0
SVM
NBayes
DTree
NN
McTwo
WRank
TRank
ROCRank

(g)

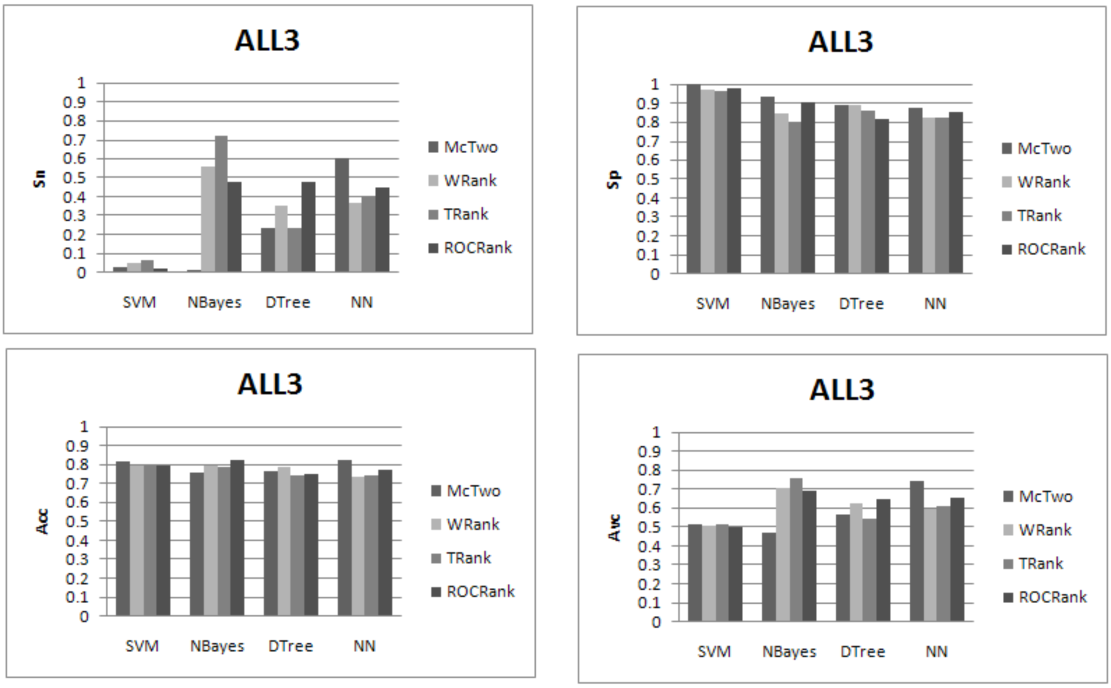
ALL3
Sn
Sp
Acc
Auc
1
0.9
0.8
0.7
0.6
0.5
0.4
0.3
0.2
0.1
0
SVM
NBayes
DTree
NN
McTwo
WRank
TRank
ROCRank

(h)

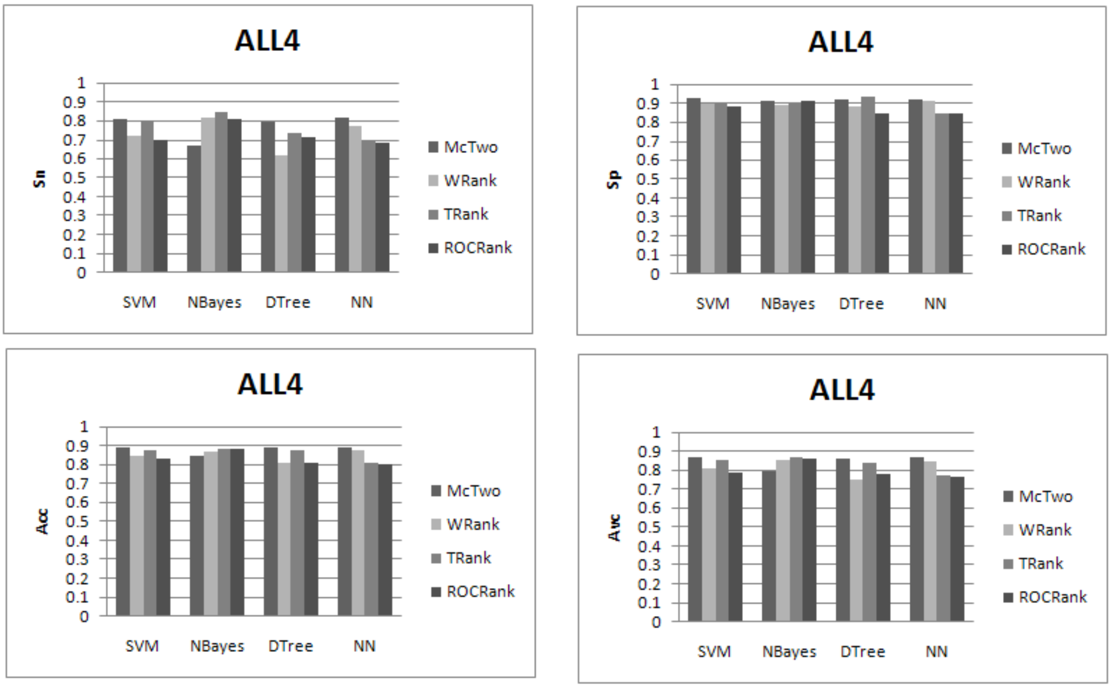
ALL4
Sn
Sp
Acc
Auc
1
0.9
0.8
0.7
0.6
0.5
0.4
0.3
0.2
0.1
0
SVM
NBayes
DTree
NN
McTwo
WRank
TRank
ROCRank

(i)

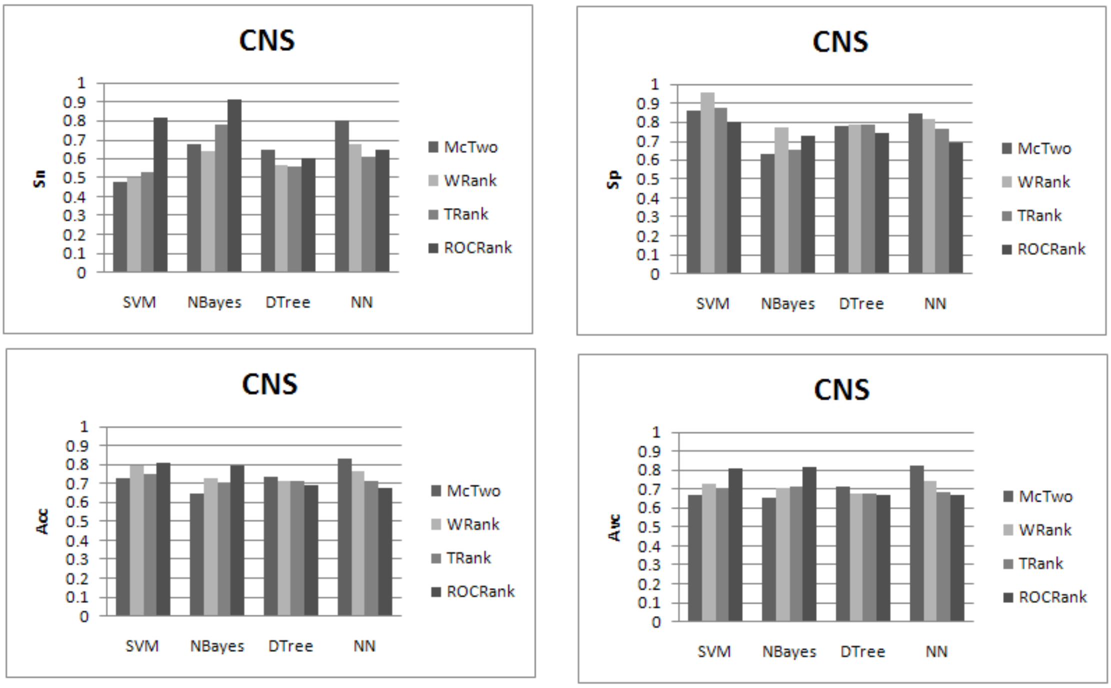
CNS
Sn
Sp
Acc
Auc
1
0.9
0.8
0.7
0.6
0.5
0.4
0.3
0.2
0.1
0
SVM
NBayes
DTree
NN
McTwo
WRank
TRank
ROCRank

(j)

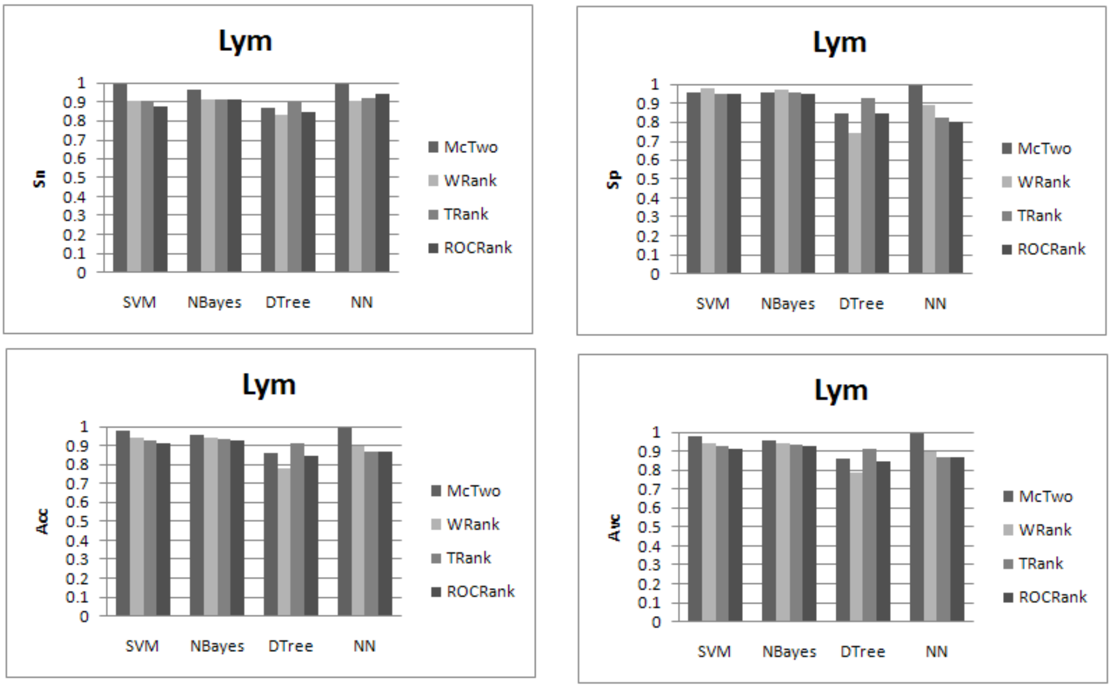
Lym
Sn
Sp
Acc
Auc
1
0.9
0.8
0.7
0.6
0.5
0.4
0.3
0.2
0.1
0
SVM
NBayes
DTree
NN
McTwo
WRank
TRank
ROCRank

(k)

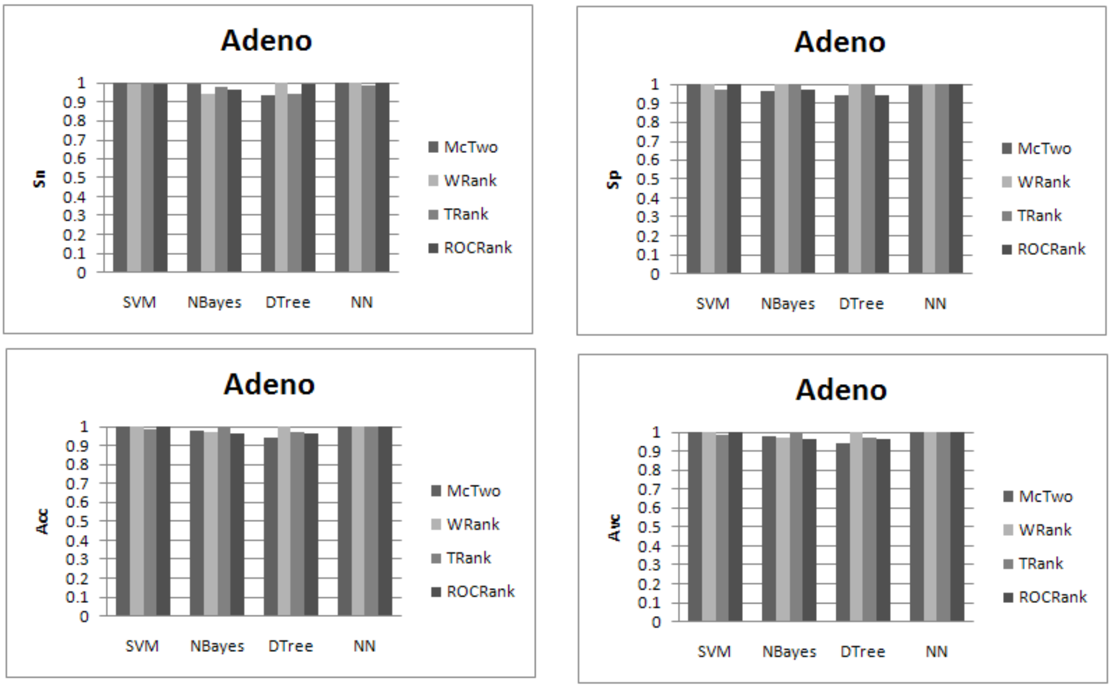
Adeno
Sn
Sp
Acc
Auc
1
0.9
0.8
0.7
0.6
0.5
0.4
0.3
0.2
0.1
0
SVM
NBayes
DTree
NN
McTwo
WRank
TRank
ROCRank

(l)

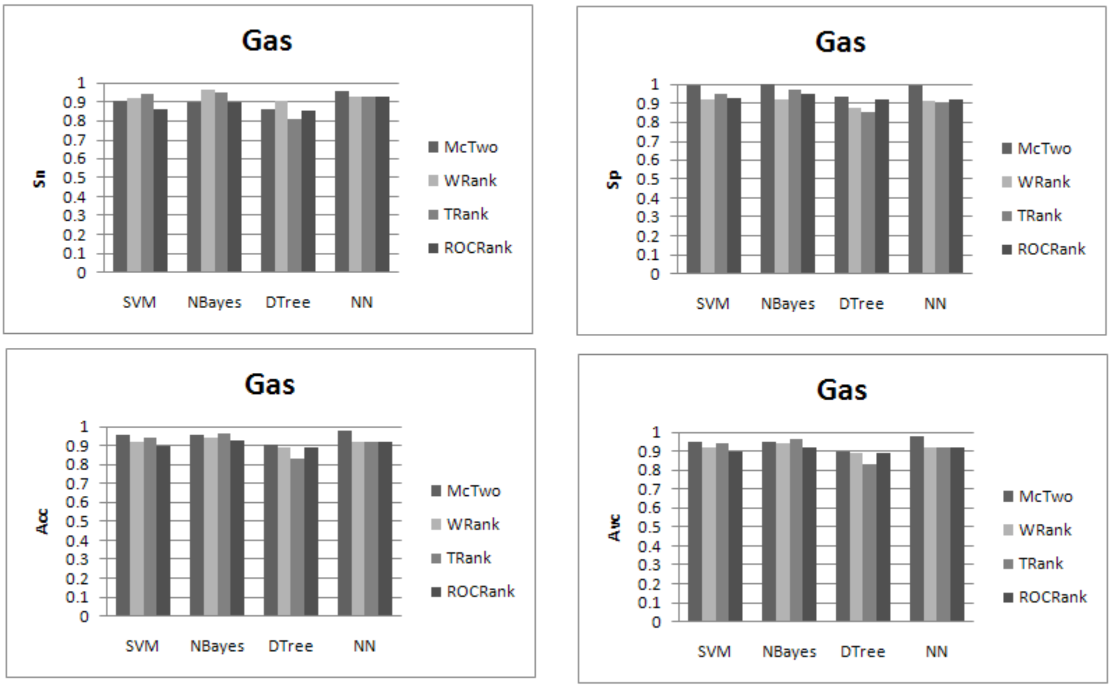

Gas
Sn
Sp
Acc
Auc
SVM
NBayes
DTree
NN
McTwo
WRank
TRank
ROCRank

(m)

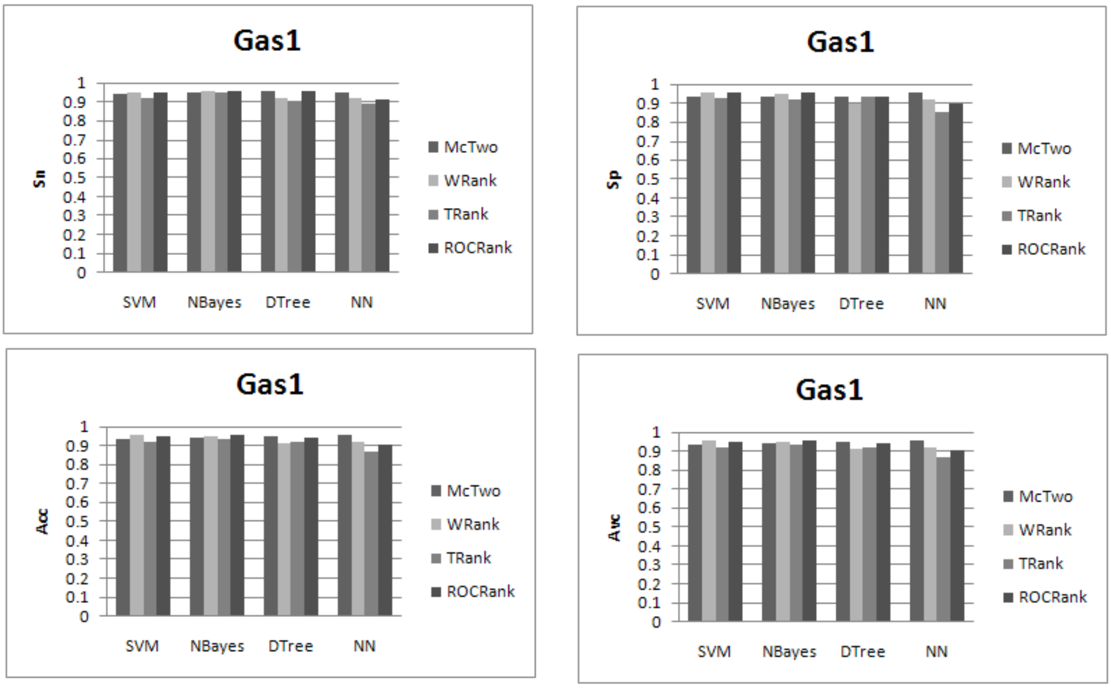

Gas1
Sn
Sp
Acc
Auc
SVM
NBayes
DTree
NN
McTwo
WRank
TRank
ROCRank

(n)

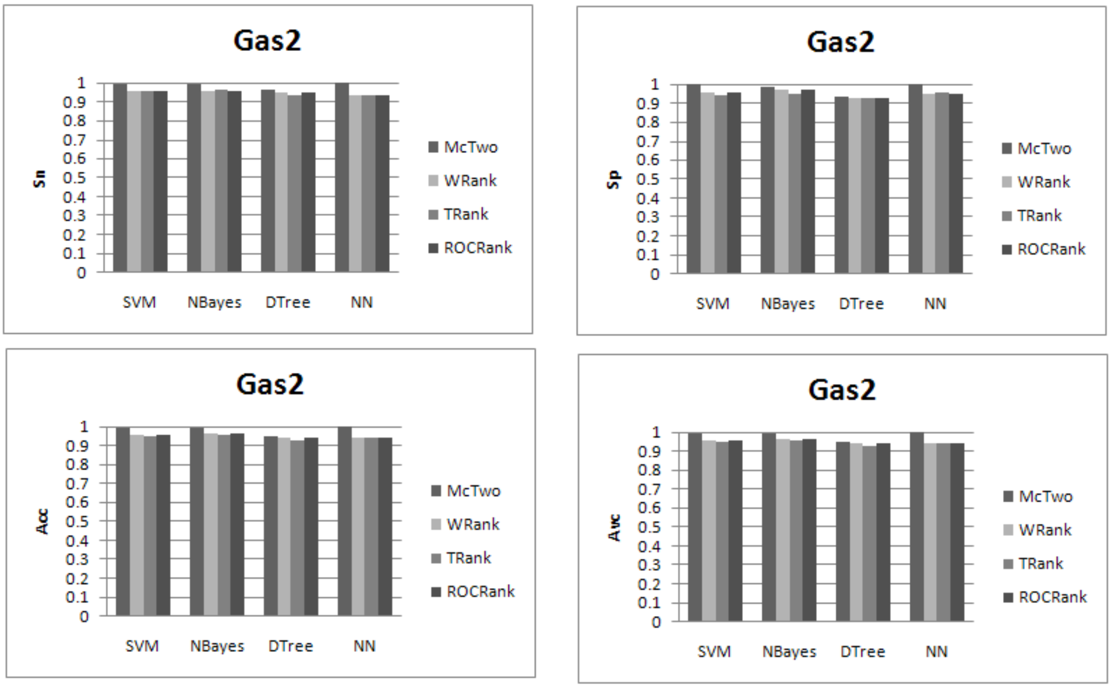
Gas2
Sn
Sp
Acc
Auc
SVM
NBayes
DTree
NN
McTwo
WRank
TRank
ROCRank

(o)

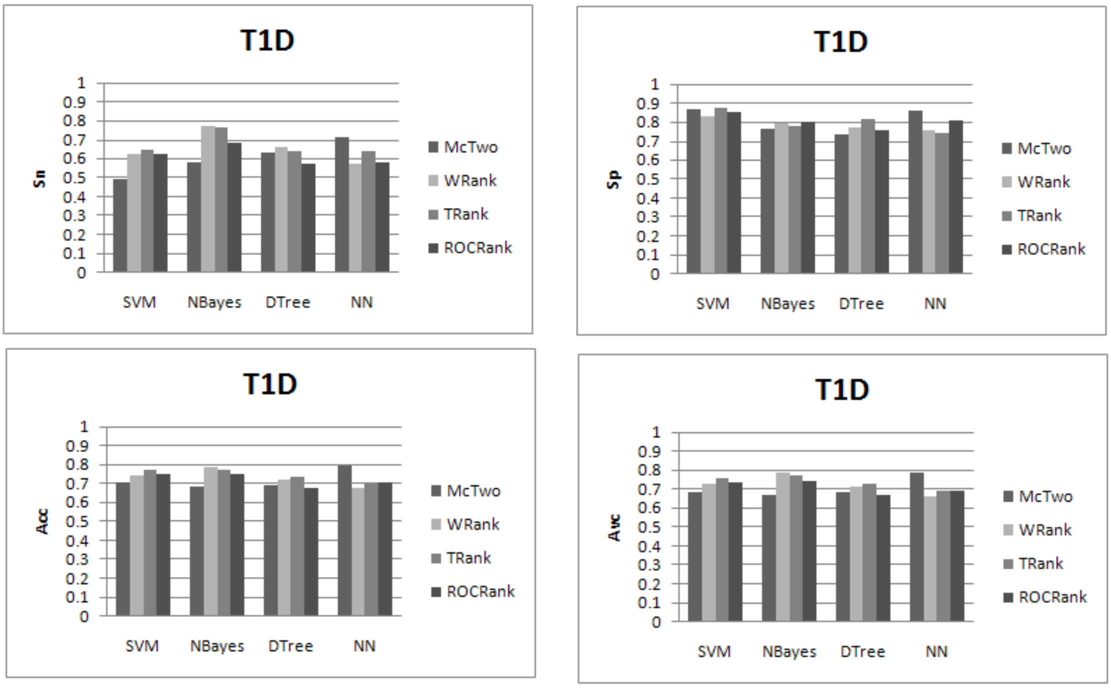
T1D
Sn
Sp
Acc
Auc
SVM
NBayes
DTree
NN
McTwo
WRank
TRank
ROCRank

(p)

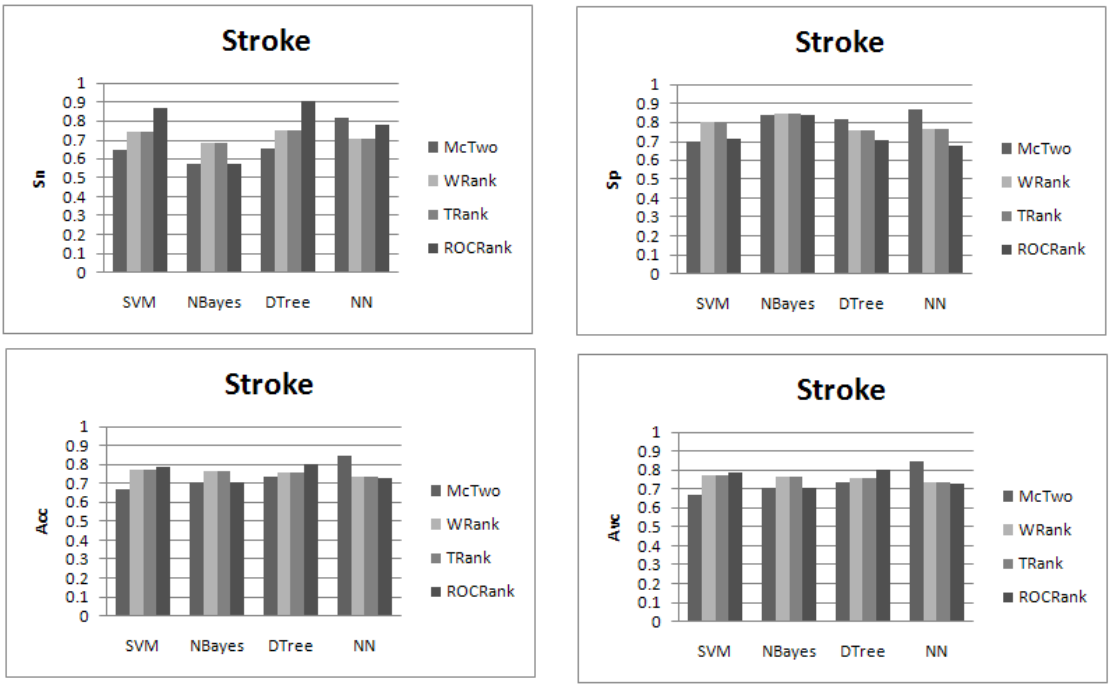
Stroke
Sn
Sp
Acc
Auc
SVM
NBayes
DTree
NN
McTwo
WRank
TRank
ROCRank
0
0.1
0.2
0.3
0.4
0.5
0.6
0.7
0.8
0.9
1

(q)

Supplementary Table S1

Comparison of the binary classification accuracy Acc between the two algorithms McTwo and McOne. The averaged value and the standard deviation of the classification mAcc are calculated over the 30 runs of the 5-fold cross validations over the 17 datasets. This table is the data for plotting the Supplementary Figure S1.

Dataset	McOne			McTwo		
	Avg(mAcc)	StdDev(mAcc)	Classifier	Avg(mAcc)	StdDev(mAcc)	Classifier
DLBCL	0.986	0.004	NBayes	0.986	0.013	NN
Pros	0.957	0.006	NBayes	0.933	0.021	NN
Colon	0.913	0.012	SVM	0.892	0.029	NN
Leuk	0.989	0.008	SVM	0.993	0.013	NN
Mye	0.859	0.012	NBayes	0.852	0.018	NN
ALL1	1.000	0/0/0	SVM/ NBayes/ NN	1.000	0/0	NN/Dtree
ALL2	0.714	0.033	NBayes	0.716	0.032	NN
ALL3	0.808	0.000	SVM	0.822	0.018	NN
ALL4	0.907	0.015	SVM	0.894	0.013	SVM
CNS	0.752	0.034	SVM	0.828	0.036	NN
Lym	1.000	0.000	NBayes	0.993	0.012	NN
Adeno	1.000	0/0	SVM/ NN	1.000	0.000	SVM
Gas	0.964	0.008	NBayes	0.977	0.014	NN
Gas1	0.972	0.000	SVM	0.953	0.011	NN
Gas2	0.992	0.000	SVM/ NBayes	0.998	0.004	NN
T1D	0.822	0.020	NBayes	0.795	0.022	NN
Stroke	0.977	0.017	NBayes	0.843	0.046	NN

Supplementary Table S2

Comparison of McTwo with the three individual ranking algorithms. The classification performance is measured in mAcc. The data highlighted in bold are the models that are better than McTwo.

mAcc	DLBCL	Pros	Colon	Leuk	Mye	ALL1	ALL2	ALL3	ALL4
McTwo	0.986	0.933	0.892	0.993	0.852	1.000	0.716	0.822	0.894
TRank	0.914	0.924	**0.903**	0.956	**0.858**	0.967	**0.759**	0.791	0.880
WRank	0.932	**0.939**	**0.903**	0.973	0.832	1.000	**0.759**	0.795	0.874
ROCRank	0.916	**0.941**	0.878	0.959	0.829	1.000	**0.749**	0.820	0.885
mAcc	CNS	Lym	Adeno	Gas	Gas1	Gas2	T1D	Stroke	
McTwo	0.828	0.993	1.000	0.977	0.953	0.998	0.795	0.843	
TRank	0.752	0.932	0.994	0.962	0.934	0.959	0.774	0.773	
WRank	0.797	0.944	1.000	0.940	0.953	0.963	0.785	0.773	
ROCRank	0.806	0.929	1.000	0.927	**0.956**	0.963	0.752	0.804	

Supplementary Table S3

Statistical significance of the comparison triplets of McTwo with the other feature selection algorithms. The comparison triplets are evaluated using paired t-test between McTwo with each of the other feature selection algorithms. The comparison triplets (win/tie/lose) are tested for their statistical significances under the confidence levels (a) 0.95, and (b) 0.99.

CT(A, B)	CFS	PAM	RRF	Rfe	FCBF	TRank	WRank	ROCRank	RfeRank
McTwo	0/0/3	0/0/3	0/3/0	0/3/0	1/2/0	0/3/0	0/3/0	0/2/1	2/1/0

(a)

CT(A, B)	CFS	PAM	RRF	Rfe	FCBF	TRank	WRank	ROCRank	RfeRank
McTwo	0/2/1	0/2/1	0/3/0	0/3/0	1/2/0	0/3/0	0/3/0	0/3/0	2/1/0

(b)
